# Engineering designer beta cells with a CRISPR-Cas9 conjugation platform

**DOI:** 10.1038/s41467-020-17725-0

**Published:** 2020-08-13

**Authors:** Donghyun Lim, Vedagopuram Sreekanth, Kurt J. Cox, Benjamin K. Law, Bridget K. Wagner, Jeffrey M. Karp, Amit Choudhary

**Affiliations:** 1 Chemical Biology and Therapeutics Science Program, Broad Institute of MIT and Harvard, Cambridge, MA 02142 USA; 2 Department of Medicine, Harvard Medical School, Boston, MA 02115 USA; 3 Divisions of Renal Medicine and Engineering, Brigham and Women’s Hospital, Boston, MA 02115 USA; 4 Engineering in Medicine, Department of Medicine, Center for Regenerative Therapeutics, Brigham and Women’s Hospital, Harvard Medical School, Boston, MA 02115 USA; 5 Harvard−MIT Division of Health Sciences and Technology, MIT, Cambridge, MA 02139 USA; 6 Proteomics Platform, Broad Institute of MIT and Harvard, Cambridge, MA 02142 USA; 7 Harvard Stem Cell Institute, Harvard University, Cambridge, MA 02138 USA

**Keywords:** Synthetic biology, CRISPR-Cas9 genome editing, Chemical tools

## Abstract

Genetically fusing protein domains to Cas9 has yielded several transformative technologies; however, the genetic modifications are limited to natural polypeptide chains at the Cas9 termini, which excludes a diverse array of molecules useful for gene editing. Here, we report chemical modifications that allow site-specific and multiple-site conjugation of a wide assortment of molecules on both the termini and internal sites of Cas9, creating a platform for endowing Cas9 with diverse functions. Using this platform, Cas9 can be modified to more precisely incorporate exogenously supplied single-stranded oligonucleotide donor (ssODN) at the DNA break site. We demonstrate that the multiple-site conjugation of ssODN to Cas9 significantly increases the efficiency of precision genome editing, and such a platform is compatible with ssODNs of diverse lengths. By leveraging the conjugation platform, we successfully engineer INS-1E, a β-cell line, to repurpose the insulin secretion machinery, which enables the glucose-dependent secretion of protective immunomodulatory factor interleukin-10.

## Abstract (web summary)

Cas9 fusions partners are often limited to natural polypeptide chains at the Cas9 termni. Here the authors present a platform for site-specific and multiple-site conjugation to both termini and internal sites of Cas9, and they apply this platform to efficiently engineer insulin-producing β cells.

## Introduction

Clustered regularly interspaced short palindromic repeats (CRISPR)-Cas9 is a DNA endonuclease that can be targeted to a genomic site using a guide RNA (gRNA) bearing sequence complementarity to the target site^1^. The genetic fusion of Cas9 with effector domains (e.g. a transcription activator) has yielded transformative technologies^2,3^; however, this approach is limited to fusions that are generally linear, polypeptidic, and located on the termini of Cas9. A covalent conjugation platform that allows the creation of fusions that are non-polypeptidic (e.g. nucleic acids, small molecules, polyethylene glycol [PEG] chains), orthogonally branched from the internal sites of Cas9, and amenable to multiple-site attachment to give a multivalent display of functional cargos would provide a greater diversity of technologies and applications. For example, precise sequence alteration at the Cas9 cleavage site requires the efficient incorporation of exogenously supplied single-stranded oligonucleotide donor DNA (ssODN)^4^ via the homology-directed repair (HDR) pathway^5,6^. However, most cells instead employ the non-homologous end-joining (NHEJ) repair, which results in unpredictable insertions and deletions of bases at the cleavage site, some of which are large enough to have pathogenic consequences^7,8^. Displaying ssODNs on Cas9 can increase their local concentration around the DNA strand break site to allow enhanced incorporation of the desired sequence. In another application, appending PEG chains to Cas9 may reduce the immunogenicity of this bacterial protein^9^.

An ideal conjugation platform to Cas9 should have the following characteristics. First, the platform should be compatible with a diverse set of cargos and allow their multiple-site attachment. Second, the platform should be robust and implementable by nonspecialists given the diverse users of CRISPR technologies. Third, since some of the cargos (e.g. ssODN) are only available in small quantities and are expensive, the conjugation system should work efficiently without requiring large excesses. Ideally, the platform should be modular and inexpensive to allow screening of multiple conditions (e.g. ssODN sequence). Finally, for real-world applications, the platform should allow scaled-up production of the conjugates following good manufacturing practice regulations^10^.

**Fig. 1:**
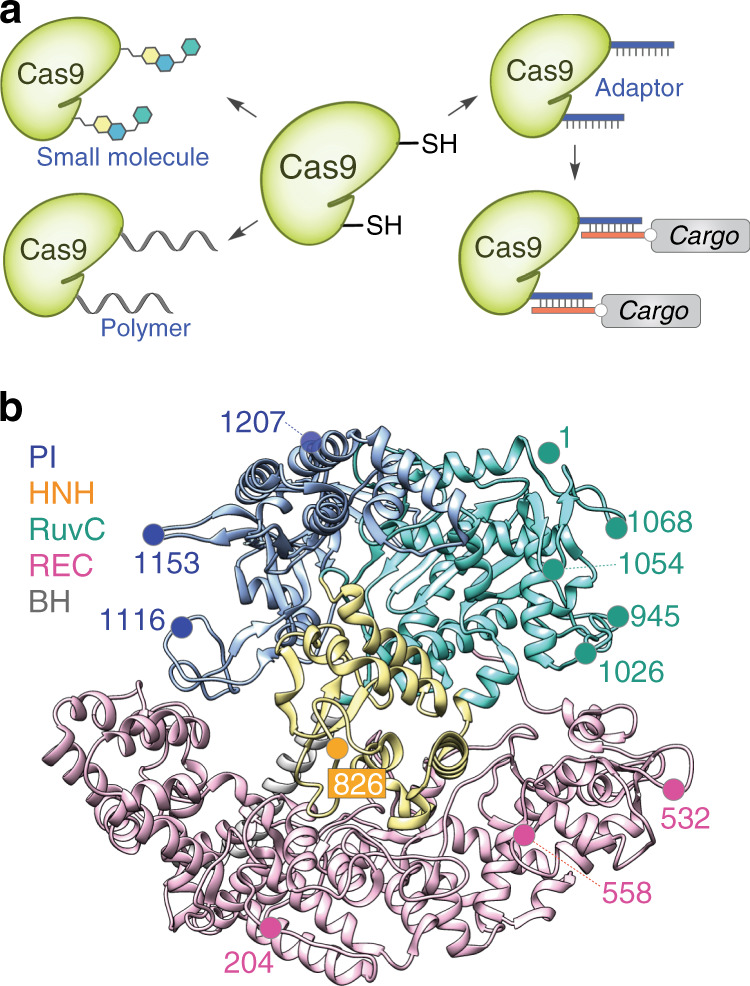
A conjugation platform for Cas9. **a** A modular design strategy to functionalize Cas9. **b** Structure-guided selection of chemical labeling sites. Protospacer adjacent motif-interacting (PI) domain is in blue, HNH domain is in yellow, RuvC domain is in cyan, recognition (REC) lobe is in magenta, and bridge helix (BH) is in grey. Crystal structure of the Cas9-gRNA-DNA ternary complex is used (PDB: 5F9R)^22^.

Herein, we present the development of such a platform that relies on thiol-maleimide chemistry and DNA-base pairing, which are both simple, well-established, scalable, and amenable to a wide range of substrates^11^. We systematically scan the domains of Cas9 to choose residues replaceable with engineered cysteines, to which molecules of any size can be efficiently appended without the loss of Cas9 activity. Because many possible conjugates (e.g. ssODNs) are prohibitively expensive for or unamenable to direct thiol-maleimide conjugation, we also seek to develop a more general conjugation platform. Thus, we design a short oligonucleotide handle ‘adaptor’, which is attached to Cas9 via thiol-maleimide chemistry and uses base pairing to anchor any molecule containing or appended with nucleic acids (Fig. 1a). As an example of the platform’s utility, we use it to hybridize long ssODNs that are large, expensive, and not available in sufficient quantities for thiol-maleimide conjugation to Cas9. The resulting Cas9:ssODN conjugates robustly enhance the precise incorporation of the desired sequence from ssODN in multiple cell types and genomic sites. Importantly, the chemical conjugation platform enables the multivalent display of ssODNs, which further enhances the precise incorporation of the desired sequence over that of the unitary display.

Next, we demonstrate the utility of our conjugation platform by efficiently engineering insulin-producing β cells to secrete nonendogenous molecules, including an immunomodulatory protein (∼160 residues), without incorporating any viral or foreign sequences (e.g. promoter) other than that of the secreted molecule. Current β-cell transplantation therapies for type 1 diabetes suffer from immune rejection, resulting in acute cell loss and only short-term therapeutic effects^12,13^. The macroencapsulation of β cells with a semipermeable membrane can protect them from the host’s immune system, although foreign body reaction-induced fibrosis can impair the mass transfer and viability of encapsulated cells^14–17^. Anti-inflammatory cytokines, such as interleukin 10 (IL-10), can reduce fibrosis and promote long-term β-cell survival and superior islet function^18–20^. Therefore, engineered β-cells that secrete anti-inflammatory cytokines and antifibrotic factors can propel the development of cell-based therapeutics for diabetes. Using our modified Cas9, we genetically repurpose the insulin expression and secretion machinery to secrete a nonendogenous peptide and IL-10 in a glucose-responsive manner, demonstrating an immediate usefulness of our genome editing platform in developing cell-based therapeutics.

## Results

### Cas9 domains tolerate the attachment of diverse molecules

To choose the sites for conjugation to Cas9, we analyzed the structures of apo-Cas9, gRNA-bound Cas9, and gRNA- and DNA-bound Cas9 for residues that could provide a high labeling yield, tolerate chemical modifications, span all the domains of Cas9, and were surface-exposed in various Cas9 conformations for the efficient modifications (Fig. 1b)^21–23^. Using the aforementioned criteria, we identified two sites (204, 532) on the recognition (REC) lobe, one site (826) on the HNH domain, five sites (1, 945, 1026, 1054, 1068) on the RuvC domain, and two sites (1153, 1207) on the protospacer adjacent motif-interacting (PI) domain. We selected residues 558 and 1116 as controls, since modifications at 558 will impede the Cas9:gRNA interaction and at 1116 will impede PAM recognition by Cas9 (Fig. 1b and Supplementary Fig. 1). We optimized the conjugation conditions for Cas9 variants using biotin-maleimide and PEG (5 kDa)-maleimide as model compounds to ensure that modifications of various sizes or natures were tolerated (Supplementary Fig. 2). The reactions were fast and high yielding at all sites except for the 1153 C mutant—sites proximal to 1153 C (i.e. 1154 C) also yielded low conjugation efficiencies (Supplementary Fig. 2). The location of these residues was not assigned at the crystal structure of apo-Cas9 but the residues were assumed to be amenable to efficient conjugation since they were expected to be surface-exposed and flexible^21–23^. Our labeling results, however, indicate that the loop may have higher-order structures that prevent efficient chemical reactions, so we did not use those sites in future experiments. To improve the compatibility of the system with a broader range of conjugates, we next utilized the optimized reaction conditions to label Cas9 at the remaining sites with a 17-nucleotide (nt) DNA adaptor (5′-GCTTCACTCTCATCGTC-3′). The conversion rates were comparable to those of PEG labeling (Supplementary Fig. 2), demonstrating that the efficient conjugation of multiple cargo types can occur at these sites. Thus, the identified sites provide high conjugation yields with diverse molecules, including small molecule and polymers (DNA or PEG).

**Fig. 2:**
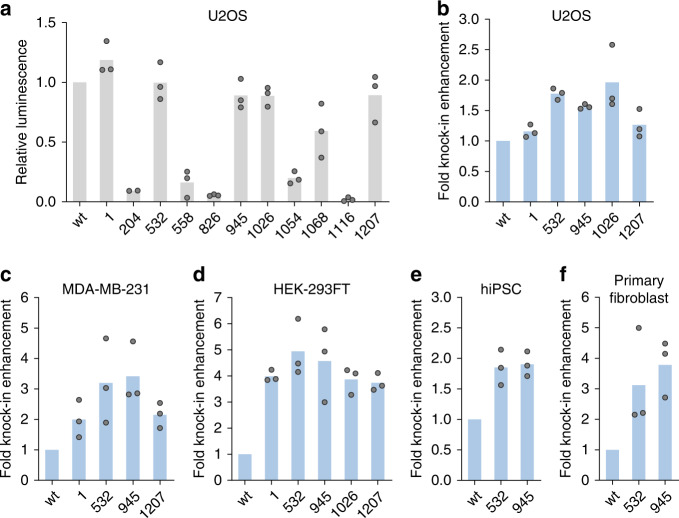
Unitary display of ssODN on Cas9 domains enhances HDR in multiple cell types. **a***HiBiT* knockin efficiencies by Cas9-adaptor conjugates compared to unlabeled wild-type Cas9 (wt) when a separate Cas9/ssODN system was used (*n* = 3 biologically independent experiments except for 204 where *n* = 2). **b**–**f** ssODN display on Cas9 enhances *HiBiT* knockin efficiency in various cells: **b** U2OS, **c** MDA-MB-231, **d** HEK-293FT, **e** human-induced pluripotent stem cells, and **f** primary human neonatal dermal fibroblasts. Unlabeled wild-type Cas9 (wt) and Cas9-adaptor conjugates labeled at the indicated residues were used (*n* = 3 biologically independent experiments). Source data are provided as a Source Data file.

To identify sites tolerant to the conjugation of the DNA adaptor without the loss of Cas9 activity, we designed an ssODN that would insert a 33-nt DNA fragment (*HiBiT* sequence^24^) at the target gene (Supplementary Fig. 3). This insertion would result in the expression of a fusion protein with a C-terminal HiBiT tag, which is a small fragment of the NanoLuc luciferase. When HiBiT is complemented by LgBiT, the remainder of NanoLuc, the full-length luciferase is reconstituted to generate a luminescence signal proportional to the degree of knockin, providing an easy readout for HDR (Supplementary Fig. 3a). We chose *GAPDH* as the first target gene (Supplementary Fig. 3b) owing to its abundant expression in many cell types, which should allow for the reliable detection of the luminescence signal. Using the *HiBiT* knockin assay, we measured whether appending the DNA adaptor to the cysteine affected Cas9 activity (Fig. 2a). As expected, much of the Cas9 activity was lost by control modifications at residues 558 and 1116, indicating the reliability of the *HiBiT* knockin assay. We identified five sites whose activity was largely maintained (>85% of wild-type in U2OS), even after labeling with the 17-nt adaptor; these sites stemmed from three regions: the REC lobe (532), the RuvC domain (1, 945, 1026), and the PI domain (1207). To investigate the off-target profile of the Cas9-adaptor conjugates, we used an *eGFP* disruption assay with matched gRNA and mismatched gRNAs targeting the *eGFP* gene of the U2OS.eGFP.PEST cells^25,26^. The Cas9-adaptor conjugate retained the target specificity while also maintaining the on-target activity (Supplementary Fig. 4a−c). Finally, we demonstrated that Cas9s conjugated to the long PEG chain (5 kDa, Supplementary Fig. 2c) retained the DNA cleavage activity in the *eGFP* disruption assay, assuring that Cas9 could be modified with diverse cargos without a loss of function (Supplementary Fig. 4d).

### Unitary display of ssODN enhances HDR in several cell types

Next, we designed ssODN with a sequence complementary to the conjugation adaptor and confirmed the binding of the ssODN bearing the complementary sequence to the Cas9-adaptor using a gel-shift assay (Supplementary Fig. 5). To measure the ability of ssODN conjugates to enhance HDR and the site dependence of such enhancements, we performed the *HiBiT* knockin assay in U2OS cells. Using the luminescence signals from unconjugated ssODN as normalization controls, we observed enhanced knockin efficiency at multiple sites (Fig. 2b and Supplementary Fig. 6a) with the ssODN attached to Cas9. We were able to confirm such enhancements in multiple cell lines, with a greater than four-fold increase in HEK-293FT cells, around a 1.9-fold increase in human-induced pluripotent stem cells, and a more than three-fold increase in primary fibroblasts (Fig. 2c−f and Supplementary Fig. 6b−e). For cells with higher HiBiT signal but lower HDR enhancements, we observed site dependence, with two internal conjugation sites (532, 945) generally performing better than the terminal conjugation site (1). An examination of the crystal structure^22^ indicates that cargos on the two internal residues are expected to align with substrate DNA, while cargos on the terminal residue project outward from the DNA, which may explain the differences in the HDR-enhancing capacities of different ssODN-bearing sites.

### ssODN display platform allows rapid and facile screening

**Fig. 3:**
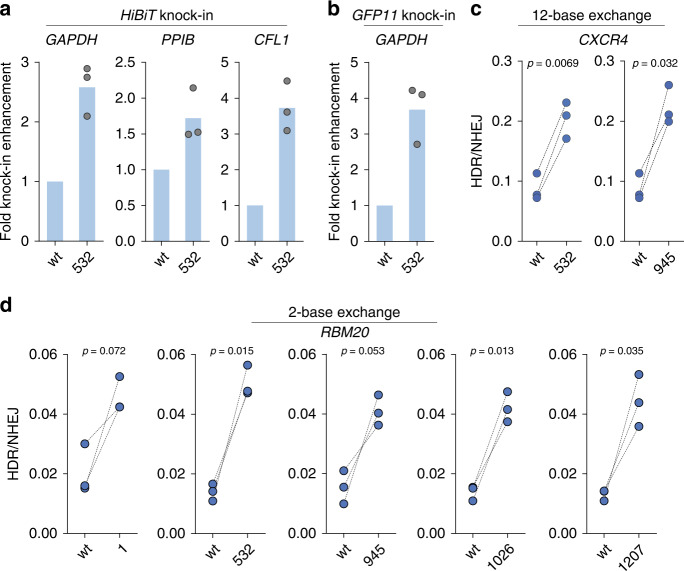
ssODN display platform allows facile testing of multiple conditions. **a** HiBiT sequence knockin efficiency was increased at multiple genomic loci in U2OS cells (*GAPDH* and *PPIB*) or HEK-293FT cells (*CFL1*), (*n* = 3 biologically independent experiments). **b** The *GFP11* sequence insertion at the *GAPDH* locus was promoted in HEK-293T cells (*n* = 3 biologically independent experiments). **c** HDR-mediated 12-base exchange efficiency at the *CXCR4* locus was increased in HEK-293T cells (*n* = 3 biologically independent experiments). **d** Two-base exchange at the *RBM20* locus was promoted in HEK-293FT cells. Unlabeled wild-type Cas9 (wt) and Cas9-adaptor conjugates labeled at the indicated residues were used (*n* = 3 biologically independent experiments). *P*-values were calculated by paired two-tailed *t*-test. Source data are provided as a Source Data file.

To demonstrate the modular nature of our conjugation platform that should allow the rapid testing of multiple conditions and to confirm the generality of HDR enhancement by ssODN display, we tested the ability of the conjugates to enhance HDR under several scenarios (e.g. different genomic sites, ssODN sequences, or readouts). Using the *HiBiT* knockin assay, we confirmed HDR enhancements at another DNA cleavage site of the *GAPDH* locus in U2OS (Fig. 3a and Supplementary Fig. 7a) and at multiple genomic loci (*PPIB* in U2OS, *CFL1* in HEK-293FT; Fig. 3a and Supplementary Fig. 7b, c). We then demonstrated HDR enhancement using a fluorescent readout and a longer knockin fragment (*GFP11*, 57 nt). The correct incorporation of this fragment generated detectable fluorescence through the expression of a fusion protein with a C-terminal GFP11 tag, which forms a fully functional GFP when complemented by GFP1-10 (Supplementary Fig. 8)^27^. Here as well, displaying ssODNs on Cas9 increased the knockin efficiency by more than three-fold (Fig. 3b and Supplementary Fig. 7d). In addition to luminescence and fluorescence readouts to demonstrate HDR enhancements, we used a restriction endonuclease site knockin assay that quantifies both NHEJ and HDR efficiencies at the *CXCR4* locus by gel electrophoresis (Supplementary Fig. 9), and observed the increase in HDR efficiencies by more than two-fold when Cas9:ssODN conjugates were employed (Fig. 3c). We then used a previously reported droplet digital PCR (ddPCR) assay that employs probes to distinguish between wild-type, NHEJ-edited, and HDR-edited sequences at the *RBM20* locus (Supplementary Fig. 10a, b)^28,29^. All Cas9:ssODN conjugates increased the ratio of HDR over NHEJ, again indicating the generality of our platform (Fig. 3d). The conjugates also enhanced HDR when another gRNA/ssODN pair was employed (Supplementary Fig. 10c). Finally, we designed an assay to convert *eGFP* to *BFP* in U2OS cells through HDR-based nucleotide exchange and found that Cas9:ssODN conjugates enhanced precision genome editing for this exogenous target gene as well (Supplementary Fig. 11).

### Multivalent display of ssODN on Cas9 further enhances HDR

**Fig. 4:**
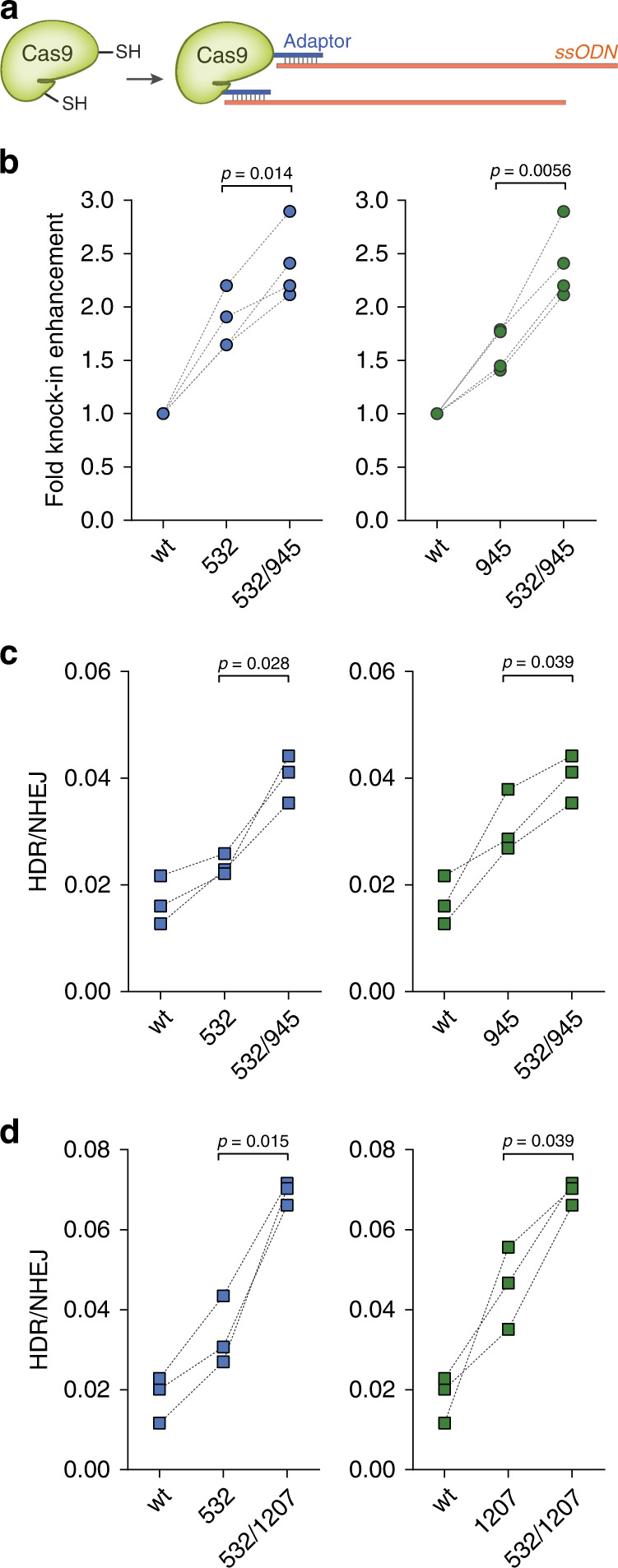
Multivalent display of ssODN further enhances HDR efficiency. **a** Schematic illustrating the production of Cas9 double-ssODN conjugates. **b***HiBiT* sequence knockin at the *GAPDH* locus was detected in U2OS cells (*n* = 4 biologically independent experiments). **c**, **d** Two-base exchange at the *RBM20* locus was detected in HEK-293FT cells. Unlabeled wild-type Cas9 (wt) and Cas9-adaptor conjugates labeled at the indicated residues were used (*n* = 3 biologically independent experiments). *P*-values were calculated by paired two-tailed *t*-test. Source data are provided as a Source Data file.

Owing to the small size of our adaptor and the chemical nature of our platform, multivalent displays are feasible (Fig. 4a). To demonstrate multivalent display, we produced Cas9 double-cysteine mutants (532 C/945 C and 532 C/1207 C) and attached the adaptor to both sites (Supplementary Fig. 12a). Next, we confirmed the binding of the ssODNs to Cas9 (Supplementary Fig. 12b) and observed a boost in HDR efficiency for both the 33-nt *HiBiT* insertion and the two-base exchange (Fig. 4b−d), indicating that multivalent Cas9 internal modifications further improve the functionality of conjugated Cas9 proteins. Finally, to minimize the size of the labels on Cas9, we investigated the possibility of further decreasing the length of the adaptor. To this effect, we found that hybridization by 13 nt or 15 nt showed a similar HDR-enhancing effect as the standard 17-nt pairing (Supplementary Fig. 13).

### Efficient engineering of β cells to secrete IL-10

**Fig. 5:**
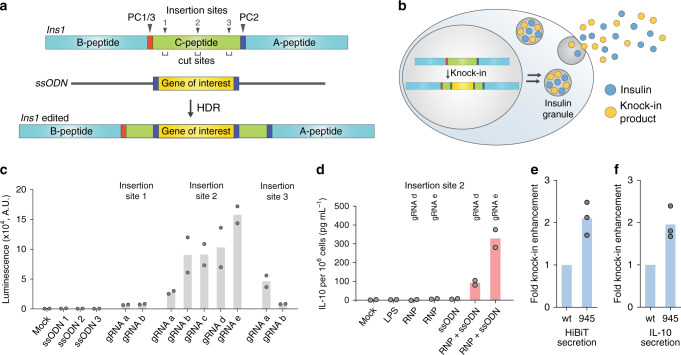
Efficient engineering of INS-1E cells for secretion of exogenous peptides and proteins. **a** Schematic of genome editing in the *Ins1* locus of INS-1E cells. PC, prohormone convertase. HDR, homology-directed repair. **b** Engineered cells can secrete exogenous gene products together with insulin. **c** INS-1E cells were engineered to secrete the 11-residue HiBiT peptide. Multiple gene insertion sites and DNA break sites were investigated (*n* = 2 biologically independent experiments). A.U. arbitrary unit. **d** INS-1E cells were engineered to secrete interleukin 10 (IL-10), (*n* = 2 biologically independent experiments). LPS lipopolysaccharide. RNP ribonucleoprotein. **e**, **f** Display of ssODN on Cas9 enhanced the secretion of **e** HiBiT peptide and **f** IL-10 (*n* = 3 biologically independent experiments). Source data are provided as a Source Data file.

To demonstrate the functional applicability of our chemically modified Cas9, we used it to efficiently engineer β cells to endow the cells with immunomodulatory function. Since C-peptide is cleaved during proinsulin processing and is cosecreted with insulin, we hypothesized that knocking in the desired gene into the C-peptide portion of the proinsulin locus would enable the secretion of the inserted gene product. Previously, a lentiviral vector encoding a proinsulin-luciferase fusion construct, containing a luciferase inserted into the C-peptide, expressed functional luciferase in levels directly proportional to insulin when stably integrated into the INS-1E rat β-cell line and responded sensitively to external stimuli, such as glucose concentration^30^. However, viral-vector engineering poses safety issues such as immunogenicity to viral components or the unintended random insertion of DNA fragments into the host genome^31,32^. Direct knockin of the desired gene fragment into the C-peptide locus using Cas9 will allow glucose-dependent cosecretion of the target gene products with insulin. The knockin strategy does not require long regulatory elements (e.g. promoters), which enables footprint-free and efficient HDR because of the smaller knockin size. Any viral or foreign sequences, which are often required to drive efficient gene expression, can be avoided to minimize the immunogenicity issues. In addition, due to the inherent high expression and secretion level of insulin in β cells, it would be easy to modulate the secretion level of exogenous gene products (Fig. 5a, b).

We set out to demonstrate our β-cell engineering strategy with the rat INS-1E β-cell line, which is widely used to study β-cell biology. Murine cells have two insulin genes, *Ins1* and *Ins2*, both of which produce and secrete functional insulin; herein, we targeted only the *Ins1* gene. To identify the appropriate insertion site in the *Ins1* locus, we used HDR-mediated knockin of the *HiBiT* sequence at the C-peptide portion (Fig. 5a). The target *HiBiT* sequence was flanked by additional prohormone convertase 2 (PC2) cleavage sites^30^ to ensure no extra amino acids would be present at each end of the knockin product after processing (Fig. 5a). We chose three gene insertion sites at the start, middle, and terminal regions of the C-peptide locus, and designed several gRNAs to target these sites such that insertion sites and DNA cleavage sites would be close enough to obtain high HDR efficiency (Fig. 5a). In addition, genome-wide off-target profiles of gRNAs were considered such that potential off-target sites had mismatches at the seed sequences or at least three mismatches in the gene-encoding regions. When standard genome editing was performed at the target sites using nonconjugated Cas9 and ssODN, the HiBiT peptide was secreted from INS-1E cells, which could be readily detected through luminescence signals from the cell culture supernatant after complementation by the LgBiT protein. The highest knockin efficiency was achieved by targeting the middle region of the C-peptide (site 2) (Fig. 5c), so this insertion site was used for future experiments. HiBiT peptide secretion was also stimulated by glucose, consistent with the behavior expected from an insertion at the *ins1* locus (Supplementary Fig. 14a).

Based on this optimized design, we next knocked in *Il10*, whose 797-nt ssODN is much larger than that of *HiBIT* (183 nt). As our approach leverages the insulin secretion pathway, the knockin product will be secreted without the secretory signal peptide. Thus, the signal peptide sequence present in the *Il10* gene was omitted when designing the knockin fragment. PC2 cleavage sites were added at each end of *Il10* to obtain intact IL-10 as the knockin product, and the corresponding ssODN was synthesized by reverse transcription. When INS-1E cells were transfected with both unconjugated Cas9 and ssODN, IL-10 was secreted to the cell culture medium as determined via enzyme-linked immunosorbent assay (ELISA). No IL-10 was detected after transfection with Cas9 or ssODN alone or in lipopolysaccharide (LPS)-treated cells^33^ (Fig. 5d). We confirmed the correct insertion of the *Il10* gene at the *Ins1* c-peptide region using Sanger sequencing (Supplementary Fig. 15a−c). Finally, we conjugated ssODN on Cas9 and found that both HiBiT secretion and IL-10 secretion were significantly promoted by Cas9-ssODN conjugation over that of separate Cas9 and ssODN (Fig. 5e, f and Supplementary Fig. 16).

**Fig. 6:**
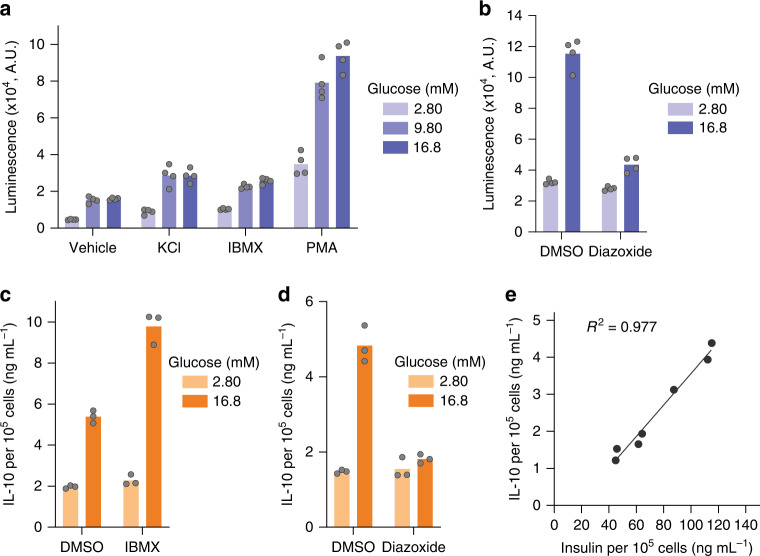
Knockin products are secreted through the insulin secretion pathway. **a**, **b** Effect of **a** known insulin secretagogues and **b** diazoxide on the HiBiT peptide secretion (*n* = 4 technical replicates). IBMX 3-isobutyl-1-methylxanthine. PMA phorbol 12-myristate 13-acetate. **c**, **d** Effect of **c** IBMX and **d** diazoxide on the IL-10 secretion (*n* = 3 technical replicates). **e** Correlation between insulin secretion and IL-10 secretion under varying glucose concentrations (from 1.40 mM to 16.8 mM). Data show means of *n* = 2 technical replicates. Source data are provided as a Source Data file.

To further verify that the knockin products are released through the regulated insulin secretion pathway, we investigated the effect of insulin secretagogues^30^ with distinct mode of actions. To minimize the well-to-well signal differences originating from the stochastic distribution of edited cells, we enriched the *HiBiT* knockin cells through sib-selection^34^, and these cells were used for the following studies. When the cells were treated with a depolarizing agent, potassium chloride, the HiBiT secretion was increased under both the low- and high-glucose conditions. The use of 3-isobutyl-1-methylxanthine (IBMX), a phosphodiesterase inhibitor that increases the intracellular cAMP level, also increased the HiBiT secretion. Phorbol 12-myristate 13-acetate (PMA), a protein kinase C activator, induced a large increase in the HiBiT secretion (Fig. 6a and Supplementary Fig. 14c). On the other hand, diazoxide, a compound that opens the potassium channel and hyperpolarizes the β cell, decreased the HiBiT secretion at the glucose-stimulated condition (Fig. 6b and Supplementary Fig. 14d). Taken together, the HiBiT signal originating from the endogenous *insulin* gene should be a reliable surrogate for insulin quantification. Due to the simplicity of HiBiT measurements compared to the insulin ELISA, our *HiBiT* knockin INS-1E cell line could be used as a reporter to study β-cell biology when high-throughput methodologies are required.

We then investigated the glucose response of IL-10 secretion after enriching the *Il10* knockin cells through sib-selection^34^. As expected from the precise insertion at the *Ins1* locus, IL-10 secretion was dependent on the glucose level (Supplementary Fig. 14b). Moreover, the secretion was stimulated by IBMX (Fig. 6c and Supplementary Fig. 14e) and was inhibited by diazoxide (Fig. 6d and Supplementary Fig. 14f). When the knockin cells were incubated with varying concentrations of glucose, insulin and IL-10 secretion was closely correlated (Fig. 6e). Genotyping and a glucose-stimulated IL-10 secretion test indicated that only 2% of the *Ins1* gene contained *Il10* knockin, though these cells still secreted on average 5.4 ng mL^−1^ of IL-10 per hour under the glucose-stimulated condition (Supplementary Fig. 14b and 15d), which is substantially higher than median effective doses of IL-10 in many in vitro assays^35^. Thus, we expect that in future therapeutic applications involving human pancreatic β-like cells, only a small portion of edited cells would need to be used to ensure normal insulin secretion is mostly intact, and it would be possible to fine-tune the secretion level of exogenous gene product.

## Discussion

We describe a simple, scalable, and modular chemical platform for site-specific Cas9 labeling with a wide range of functional molecules to expand Cas9 functionality. We first identified multiple internal residues on Cas9 that can be modified without compromising Cas9 activity in cells, and our use of thiol-maleimide chemistry opens up a variety of site-specific conjugation locations with simple and easy-to-use chemistries. We showed that internal conjugation sites and multivalent conjugations often had improved knockin efficiencies, indicating the need for conjugation systems that are not limited to single modifications of the Cas9 termini. The identified sites could also be used to attach inhibitors of DNA repair pathways for their local inhibition at the site of the Cas9-induced double-strand break. For example, the coadministration of Cas9 with small-molecule inhibitors of the NHEJ pathway can enhance precision editing^36^, but concerns about mutagenesis stemming from genome-wide NHEJ inhibition has limited the utility of such inhibitors^37,38^. Local NHEJ-pathway inhibition at the strand break site through the multivalent display of NHEJ inhibitors on Cas9 itself may allay such concerns. Such local inhibition has been demonstrated for base editors that display peptidic inhibitors of uracil DNA glycosylase for local enzyme inhibition, which improves base-editing efficiencies^39^. Similarly, the local inhibition of p53 pathway activation can increase the efficiency of precision genome editing in primary cells and stem cells where Cas9-induced double-strand breaks lead to apoptosis via this pathway^40,41^. Finally, displaying tissue-specific ligands on Cas9 will enable cell-specific genome editing^42^.

We also developed a short oligonucleotide handle as a universal anchoring point for any oligonucleotide-containing functional molecules, making this platform amenable to most desired conjugates. When ssODN was attached to this anchor, HDR efficiency was enhanced in multiple cells and genomic loci, simultaneously demonstrating the utility of the conjugation technique as well as the usefulness of an increased local concentration of ssODNs for precision genome editing. Our Cas9-adaptor design is modular in that the Cas9 bearing the universal adaptor can be used for any ssODN or any type of knockin (short nucleotide exchange, short DNA insertion, and long gene insertion). The use of the optimal ssODN sequence is crucial for successful HDR-based genome editing^6^, and the modular nature of our conjugation platform would allow rapid screening of diverse ssODNs to identify the best one. Even though absolute editing efficiencies are low in some examples (Supplementary Table 7), screening of various ssODN sequences combined with gRNA optimization and modulation of the DNA repair pathways^36^ would enable efficient HDR for practical applications. Finally, the conjugation of ssODNs to Cas9 did not negatively affect cell proliferations, ensuring the usefulness of our conjugation strategy (Supplementary Fig. 17).

While these studies were underway, reports of genetic fusions of Cas9 to avidin, SNAP tag, or porcine circovirus 2 protein (PCV), which can also bind to donor DNAs, appeared in the literature^43–46^, and our studies complement these approaches in multiple ways. First, at 17-nt, our Cas9-adaptor constructs are much smaller than the reported constructs, with the possibility of reducing this to as low as 13 nt. Second, while these other genetic fusions are mostly tested at the N- and C-termini of Cas9, we systematically investigated both terminal and internal conjugation sites and found that internal sites yielded higher knockin efficiencies in certain cell lines. The ability of our system to equally address internal or terminal sites makes it significantly more flexible and adaptable to specific applications. Third, our adaptor-based conjugation strategy does not require chemical modification of the ssODNs, as opposed to avidin- or SNAP-based methods, which can be particularly costly and time-consuming when multiple ssODNs or conditions are being screened during optimization. Fourth, our adaptor sequence can be readily altered to prevent secondary structure formation depending on the ssODN sequence, while the PCV recognition sequence cannot be changed. Finally, owing to the small size of our adaptor and the chemical nature of our platform, multivalent displays are feasible that further enhance HDR and can open up unexplored applications for conjugated Cas9 proteins, expanding the possible range of genome engineering technology.

Strategies for β-cell genome editing are urgently needed for endowing the cells with required functions, including immunomodulation, to propel the development of cell-based therapeutics for type 1 diabetes^14–17^. Using CRISPR-Cas9 and HDR-based genome editing, we demonstrated that INS-1E cells can be precisely engineered to secret a diverse set of functional molecules, such as small peptides and large gene products. Specifically, we successfully produced cells that cosecrete IL-10, a well-established anti-inflammatory factor that reduces fibrosis and can protect β cells from proinflammatory cytokine-induced cell death^18–20,47^. This method should also enable the continuous local production of immunomodulatory factors in β cells for preventing β-cell failure, yet due to its local nature, have minimal systematic effects on the host immune system^48^. Since insulin is secreted in large quantities by β cells, only a small fraction of cells may require editing to have a therapeutic effect. Our approach of repurposing the insulin expression and secretion machinery significantly reduces the size of the exogenous sequences that need to be knocked in, allowing efficient engineering of the β cells using Cas9. Moreover, our precise knockin strategy would be safer than conventional random gene integration methods, which use immunogenic viral vectors that result in unpredictable genomic sequences^31,32^. Because the knockin site was located at the c-peptide region, the knockin product was secreted in a glucose-dependent manner, and the secretion level was sensitive to insulin secretagogues. Glucose responsiveness would be advantageous for cells engineered to secrete glucagon-like peptide 1 receptor agonists, which when cosecreted with insulin, would promote insulin secretion and enhance the viability of β cells^49^. Additionally, many autoantigens in type 1 diabetes originate from the insulin granule, which may require enhanced immunomodulatory functions under high-glucose conditions^50^.

Overall, this study provides a simple and effective method for forming chemical conjugates of Cas9 to enhance its functionality, and its ease-of-use will make it convenient for scientists of all backgrounds to modify existing Cas9 tools to suit their desired applications. The example application of our method shows that Cas9:ssODN conjugates can successfully enhance precision genome editing in β cells, opening up possibilities for chemically enhanced Cas9 in regenerative medicine.

## Methods

### Cas9 expression and purification

A plasmid for SpCas9 expression (2x NLS and C-terminal His tag, pET-28a) was a gift from the Gao group (Addgene #98158)^51^. *E. coli* Rosette2 (DE3) expressing wild-type Cas9, single-cysteine Cas9 mutants, or double-cysteine Cas9 mutants were grown overnight at 18 °C with 0.5 mM of IPTG supplemented when the OD_600 nm_ reached 0.8–1.2. The protein was purified by successive Ni-NTA affinity chromatography, cation exchange chromatography, and size-exclusion chromatography. Purified proteins were snap-frozen in liquid nitrogen and stored at −80 °C in Cas9 storage buffer (20 mM Tris·HCl, 0.1 M KCl, 1 mM TCEP, 20% glycerol, pH 7.5).

### Site-directed mutagenesis

Two cysteine residues in SpCas9 (C80, C574) were replaced by serine to give a cysteine-free mutant. Based on this construct, multiple single-cysteine and double-cysteine mutants were generated by introducing cysteines at the designated residues. Mutagenesis was performed using the partial overlapping primer design method or using a Q5 Site-Directed Mutagenesis Kit (New England Biolabs). UCSF Chimera 1.13 was used to analyze the protein structures and choose the mutation sites.

### Cas9 labeling by thiol-maleimide conjugation

Adaptor oligonucleotide (GCT TCA CTC TCA TCG TC) modified with protected maleimide (maleimide-2,5-dimethylfuran cycloadduct) at the 5’ terminus was synthesized by Gene Link. Prior to thiol-maleimide conjugation, the maleimide group was deprotected via retro-Diels-Alder reaction by heating the DNA in toluene for 3 h at 90 °C. Solvent was removed under the reduced pressure, and the resulting DNA in solid form was dissolved in water to give 2 mM solution. Cas9 cysteine mutants (4 μM) were mixed with 300 *μ*M of PEG (5 kDa)-maleimide or adaptor oligonucleotide-maleimide in reaction buffer (20 mM Tris·HCl, 0.1 M KCl, 1 mM TCEP, pH 7.5). The reaction proceeded for 3 h at room temperature (RT) with mild rotation. The resulting mixture was diluted with a high-salt buffer (20 mM Tris·HCl, 1 M KCl, 1 mM TCEP, 20% glycerol, pH 7.5) and incubated with Ni-NTA agarose beads at 4 °C. The beads were extensively washed with the high-salt buffer to completely remove non-specifically bound oligonucleotide molecules. Labeled Cas9 was eluted with an elution buffer (20 mM Tris·HCl, 0.1 M KCl, 1 mM TCEP, 250 mM imidazole, 10% glycerol, pH 7.5). Finally, buffer exchange was conducted using Amicon Ultra-0.5 mL centrifugal filters with a 100 kDa cut-off (Millipore) to give Cas9-adaptor conjugates in storage buffer (20 mM Tris·HCl, 0.1 M KCl, 1 mM TCEP, 10% glycerol, pH 7.5).

### Cas9 biotin labeling and pull-down by streptavidin beads

Cas9 with enhanced specificity [eSpCas9(1.1)]^52^ was used for biotin labeling. Cas9 cysteine mutants (7 μM) were mixed with 500 μM of EZ-Link™ Maleimide-PEG_2_-Biotin (Thermo) in a reaction buffer (20 mM Tris·HCl, 0.1 M KCl, 1 mM TCEP, pH 7.5). The reaction proceeded for 4 h at room temperature (RT) with mild rotation. Excess compounds were removed by Bio-Gel P-6 columns (Biorad) according to the manufacturer’s protocol. Next, 30 pmol of Cas9 from the above step was incubated with 30 μL of Pierce Streptavidin Magnetic Beads (Thermo) overnight at 4 °C. Flow-through was collected and the beads were washed twice with a washing buffer (20 mM Tris·HCl, 0.15 M NaCl, 0.1% Tween20, pH 7.4; 300 μL) and once with the reaction buffer (300 μL). The beads were heated to 95 °C for 5 min in the presence of SDS-PAGE buffer, and the resulting bead-bound fraction (eluate) and flow-through were subjected to SDS-PAGE followed by Coomassie staining.

### Electrophoretic mobility shift assay

For this assay, 300 nM of Cas9 was mixed with 300 nM of ssODNs in a binding buffer (20 mM Tris·HCl, 0.1 M KCl, 1 mM TCEP, 10% glycerol, pH 7.5). For the Cas9 double-adaptor conjugates, 200 nM of protein and 400 nM of ssODN were used. For testing long ssODNs (Supplementary Fig. 16e), 80 nM Cas9 and 60 nM ssODN were used. The mixture was incubated for 30 min at RT and resolved by 1% agarose gel. DNA was stained using SYBR Gold, and fluorescence images were obtained using an Azure c600 (Azure Biosystems) with the Cy3 channel.

### In vitro transcription to synthesize single-guide RNAs

Sequences of target-specific forward primers and universal reverse primers are listed in Table S2. Polymerase chain reactions (PCRs) were conducted using Q5 High-Fidelity 2× Master Mix (New England Biolabs) in the presence of 0.5 μM of forward and reverse primers in a volume of 25 μL. The PCR program was as follows: Initial denaturation at 95 °C for 1 min; 25 cycles of 95 °C for 15 s, 58 °C for 30 s, and 72 °C for 15 s; final extension at 72 °C for 2 min and cooling to 25 °C using a 1% ramp. The resulting mixture was used for in vitro transcription without purification. The reaction was performed using the HiScribe T7 Quick High Yield RNA Synthesis Kit (New England Biolabs). The mixture contained 10 μL of NTP buffer mix, 2 μL of the above crude PCR product, 2 μL of T7 RNA polymerase mix, and 0.75 μL (30 U) of recombinant RNase inhibitor (New England Biolabs) in a final volume of 30 μL. The reaction was conducted for 12 h at 37 °C. DNase treatment was performed to remove template DNA according to the manual. RNAs were purified using the MEGAclear Transcription Clean-Up Kit (Invitrogen) according to the manual.

### Short single-stranded oligonucleotides

Single-stranded donor DNAs for *HiBiT* insertion, *GFP11* insertion, and nucleotide exchange at the *CXCR4* and *RBM20* locus were Ultramer DNA oligonucleotides synthesized by Integrated DNA Technology. Their sequences are listed in Supplementary Table 3.

### Long single-stranded oligonucleotides for *Il10* insertion

Single-stranded donor DNAs for *Il10* insertion were synthesized by reverse transcription^53^. First, double-stranded gBlocks DNAs were synthesized by Integrated DNA Technology. The DNAs have the T7 promoter sequences followed by the reverse complementary sequences of the final ssODN sequences. DNAs were produced in large quantities by PCR, followed by gel electrophoresis and gel extraction. Next, in vitro transcription was performed using the HiScribe T7 Quick High Yield RNA Synthesis Kit (New England Biolabs). The mixture contained 10 μL of NTP buffer mix, 400 ng of the double-stranded DNA template, 2 μL of T7 RNA polymerase mix, and 0.4 μL (16 U) of recombinant RNase inhibitor (New England Biolabs) in a final volume of 20 μL. The reaction was conducted for 4 h at 37 °C. DNase treatment was performed to remove template DNA according to the manual. The resulting RNAs were purified using the MEGAclear Transcription Clean-Up Kit (Invitrogen) according to the manual. Finally, reverse transcription was performed to obtain single-stranded donor DNAs. Approximately 200–250 pmol of RNA was mixed with 400 pmol of reverse primer and 6 μL of dNTP mix (25 mM each, New England Biolabs) in nuclease-free water at a final volume of 35 μL. The mixture was incubated at 65 °C for 5 min, then immediately placed on ice for 5 min to induce RNA-primer annealing. Then, 10 μL of 5× RT buffer (250 mM Tris·HCl, 375 mM KCl, 15 mM MgCl2, pH 8.3), 2.5 μL of 0.1 M dithiothreitol solution, 0.5 μL (20 U) of RNase inhibitor (New England Biolabs), and 2.5 *μ*L of TGIRT-III reverse transcriptase (InGex) were added to the RNA-primer solution. The reaction was proceed at 58 °C for 3 h. Next, RNA was hydrolyzed by adding 21 μL of 0.5 M NaOH solution and heating at 95 °C for 10 min. The basic solution was quenched by the addition of 21 μL of 0.5 M HCl solution. The resulting single-stranded DNAs were purified using MinElute PCR Purification Kit (Qiagen) according to the manual. The purity of the ssDNA was confirmed by 6% TBE-Urea gel electrophoresis followed by SYBR Gold staining. All DNA sequences are listed in Supplementary Tables 4 and 5.

### Cell culture

U2OS, MDA-MB-231, HEK-293T, HEK-293FT, and human dermal fibroblasts neonatal (HDFn) cells were maintained in Dulbecco’s Modified Eagle Medium (DMEM) supplemented with 10% fetal bovine serum, 1 mM pyruvate, and penicillin-streptomycin. Human-induced pluripotent stem cells (hiPSC) were maintained on Matrigel in E8 medium. When single-cell passaging of the hiPSC was required, Y-27632 was supplemented at a final concentration of 10 μM. INS-1E cells were maintained in Roswell Park Memorial Institute (RPMI) medium supplemented with 10% fetal bovine serum, 1 mM pyruvate, penicillin-streptomycin, and 50 μM of 2-mercaptoethanol.

### *eGFP* disruption assay

Cas9 (10 pmol) and gRNA (10 pmol) were mixed and incubated at RT for 5 min. U2OS.eGFP.PEST cells were transfected with the ribonucleoprotein (RNP) complex using the SE Cell Line 4D-Nucleofector kit (Lonza) following the pulse program of DN-100. After transfection, the cells were suspended in the culture media and transferred to a 96-well plate (20,000 cell per well). Forty-eight hours after transfection, the cells were fixed with a 4% paraformaldehyde solution, and the nuclei were stained with HCS NuclearMask Blue Stain (Invitrogen). The resulting fluorescence signals from eGFP and nuclei were measured using an ImageXpress Micro High-Content Analysis System (Molecular Devices) or an Operetta CLS High-Content Analysis System (PerkinElmer). Data acquisition and analysis was performed using MetaXpress (Molecular Devices) or Operetta Harmony 4.8 (PerkinElmer).

### *HiBiT* sequence knockin by nucleofection

U2OS cells stably expressing eGFP.PEST, MDA-MB-231 cells, hiPSC, and HDFn were transfected with Cas9 RNP and ssODN using the cell-specific 4D-Nucleofector kit (Lonza). For U2OS cells, the SE Cell Line kit and the program DN-100 were used. For MDA-MB-231 cells, the SE Cell Line kit and the program CH-125 were used. For hiPSC, the P3 Primary Cell kit and the program CA-137 were used. For HDFn, the P3 Primary Cell kit and the program EN-150 were used. For U2OS and MDA-MB-231 cells, 10 pmol of Cas9, gRNA, and ssODN were used. For hiPSC and HDFn, 20 pmol of Cas9, gRNA, and ssODN were used. For the Cas9:ssODN conjugates, the Cas9-adaptor was premixed with ssODN and incubated at RT for 15–30 min prior to RNP formation to ensure Cas9:ssODN conjugate formation. Then gRNA was added, and the final mixture was incubated for 5–10 min at RT. When Cas9 would not specifically bind ssODNs, the RNP was formed first because it is known that nonspecific Cas9-DNA interactions hamper the RNP formation. After incubating Cas9 and gRNA at RT for 5–10 min, ssODN was added to the mixture. Approximately 200,000 cells (U2OS and MDA-MB-231) or 500,000 cells (hiPSC and HDFn) were transfected with the above mixtures in a well of the nucleofection kit, and 20,000 transfected cells were seeded in each well of a 96-well plate in the case of U2OS and MDA-MB-231 cells. For hiPSC and HDFn, all the cells were plated in a well of a 24-well plate. Cells were incubated for 24 h (U2OS and MDA-MB-231) or 48 h (hiPSC and HDFn) at 37 °C, and cell viability was measured using the PrestoBlue Cell Viability Reagent (Thermo) with SpectraMax M5 and SoftMax Pro 7.0 (Molecular Devices). Next, the HiBiT detection was performed using the Nano-Glo HiBiT Lytic Detection System (Promega) according to the manufacturer’s protocol. The luminescence signal was recorded by an EnVision Multilabel Plate Reader with EnVision Manager 1.13 (PerkinElmer). The resulting luminescence signals were normalized based on the cell viability. The same instruments and software were used for all the following *HiBiT* knockin assays.

### *HiBiT* sequence knockin by lipofection

HEK-293FT cells were seeded in a 96-well plate at a density of 10,000 cells per well. The next day, Lipofectamine CRISPRMAX (Invitrogen) was used to transfect the cells with Cas9 RNP and ssODN, with final concentrations of 25 nM of both reagents in 110 μL of medium per well in a 96-well plate. For Cas9:ssODN conjugates, the Cas9-adaptor was premixed with the ssODN in Opti-MEM (Gibco) and incubated at RT for 15–30 min prior to RNP formation. Next, gRNA was added, and the mixture was incubated for 5–10 min at RT. Then, Plus reagent (Thermo; 0.17 μL per well) was added, and the mixture was incubated for an additional 5 min. Finally, Lipofectamine CRISPRMAX (0.3 μL per well) in Opti-MEM was added, and the mixture was incubated at RT for 5 min. The final transfection mixture was transferred to each well. In cases where Cas9 did not specifically bind ssODNs, the RNP was first formed by incubating Cas9 and gRNA at RT for 5–10 min in Opti-MEM. Then, Plus reagent (0.17 μL per well) was added, and the mixture was incubated for an additional 5 min. Next, the ssODN was added to the mixture. Finally, Lipofectamine CRISPRMAX (0.3 μL per well) in Opti-MEM was added, and the mixture was incubated at RT for 5 min. The final transfection mixture was transferred to each well. The transfections were performed in three technical replicates for each biological replicate. For knockin experiments using Cas9 double-ssODN conjugates (Fig. 4), RNP formation was performed first to prevent the nonspecific Cas9-ssODN interaction that blocks RNP formation and decreases the genome editing efficiency. Cells were incubated for 24 h at 37 °C, and cell viability was measured using the PrestoBlue Cell Viability Reagent. Next, the HiBiT detection was performed using the Nano-Glo HiBiT Lytic Detection System. The resulting luminescence signals were normalized based on the cell viability.

### *GFP11* sequence knockin by lipofection

HEK-293T cells were seeded in a 96-well plate at a density of 8000 cells per well. The next day, Lipofectamine CRISPRMAX was used to transfect the cells with Cas9 RNP (30 nM) and ssODN (30 nM) using the same procedures as described above for the *HiBiT* knockin assay. Approximately 20–22 h post transfection, the media was exchanged, and the cells were incubated for an additional 2–4 h. Then, a plasmid encoding for a *GFP1-10* fragment (Addgene #70219, a kind gift from Prof. Bo Huang)^27^ was delivered to the cells using Lipofectamine 2000 (Invitrogen) (120 ng plasmid and 0.4 μL Lipofectamine per well). A total of 50 h after RNP and ssODN transfection, the cells were fixed using 4% paraformaldehyde. Nuclei were stained by HCS NuclearMask Blue Stain (Invitrogen), and fluorescence images were obtained using the ImageXpress Micro (Molecular Devices) with the DAPI and GFP channels. Data acquisition and analysis was performed using MetaXpress (Molecular Devices).

### Knockin of the restriction endonuclease site

HEK-293T cells were seeded in a 24-well plate at a density of 80,000 cells per well. The next day, Lipofectamine CRISPRMAX was used to transfect the cells with Cas9 RNP (50 nM) and ssODN (50 nM) using the same procedures as described above for the *HiBiT* knockin, with 1.5 μL of Plus reagent and 2.5 μL of CRISPRMAX in a final volume of 550 *μ*L per well. Forty-eight hours post transfection, genomic DNA was extracted using the DNeasy Blood & Tissue Kit (Qiagen). The *CXCR4* target site was amplified by a primer pair listed in Supplementary Table 6 with the annealing temperature of 67 °C and the extension time of 35 s using Q5 Hot Start High-Fidelity 2× Master Mix (New England Biolabs). The PCR product was purified using the MinElute PCR Purification Kit (Qiagen). The HDR efficiency was measured by HindIII digestion of 100 ng of PCR product with 10 units of HindIII-HF (New England Biolabs) in CutSmart Buffer for 1 h at 37 °C. The reaction products were resolved using 1.2% agarose gel containing SYBR gold (Invitrogen), and gel images were obtained using an Azure c600 (Azure Biosystems). The band intensities were quantified with ImageJ 1.52a, and the percentage of HDR was estimated using the following formula: 100 × (*b* + *c*)/(*a* + *b* + *c*), where *a* is the intensity for the undigested PCR product, and *b* and *c* are the intensities for the HindIII cleavage products. NHEJ efficiency was measured by T7EI assay using T7 Endonuclease I (New England Biolabs) based on the methods suggested by the supplier. The reaction products were resolved using 1.2% agarose gel containing SYBR gold, gel images were obtained using an Azure c600, the band intensity was quantified with ImageJ 1.52a, and the percentage of NHEJ was calculated using the following formula: 100 × {1 – [1 – (*b* + *c*)/(*a* + *b* + *c*)]^1/2^}, where *a* is the intensity for the undigested PCR product, and *b* and *c* are the intensities for the T7E1 cleavage products.

### Droplet digital PCR-based assay to quantify NHEJ and HDR

HEK-293FT cells were seeded in a 96-well plate at a density of 10,000 cells per well. The next day, the cells were transfected with Cas9 RNP (35 nM) and ssODN (35 nM) using Lipofectamine RNAiMAX (Invitrogen) in 110 μL of media per well in a 96-well plate. For Cas9:ssODN conjugates, the Cas9-adaptor was premixed with ssODN in Opti-MEM (Gibco) and incubated at RT for 15–30 min prior to RNP formation. Next, gRNA was added, and the mixture was incubated for 5–10 min at RT. Finally, Lipofectamine RNAiMAX (0.3 μL per well) in Opti-MEM was added, and the mixture was incubated at RT for 5 min. The final transfection mixture was transferred to each well. In cases where Cas9 does not specifically bind to the ssODNs, the RNP was first formed by incubating Cas9 and gRNA at RT for 5–10 min in Opti-MEM. Then, the ssODN was added to the mixture. Finally, Lipofectamine RNAiMAX (0.3 μL per well) in Opti-MEM was added, and the mixture was incubated at RT for 5 min. The final transfection mixture was transferred to each well. For experiments using Cas9 double-ssODN conjugates (Fig. 4), RNP formation was performed first because more ssODN was used in comparison to RNP. Two days post transfection, the cells were harvested, and the genomic DNA was extracted using a DNeasy Blood & Tissue Kit (Qiagen). Genomic sequences were read by droplet digital PCR^29^. The PCR premixture contained 10 μL of ddPCR Supermix for Probes (No dUTP) (Bio-Rad #1863024), 100 ng of genomic DNA, 900 nM of forward and reverse primers each, 250 nM of reference probe, 250 nM of HDR probe, 250 nM of NHEJ probes, 500 nM of dark probe, and 8 U of HindIII-HF (New England Biolabs) in a 20 μL solution. Droplets were generated from the premixture and Droplet Generation Oil for Probes (Bio-Rad) using a QX200 Droplet Generator (Bio-Rad) according to the manufacturer’s instruction. Next, droplets were subjected to standard PCR using an Eppendorf Mastercycler Pro thermal cycler with the following program: 95 °C for 10 min (×1); 94 °C for 30 s and 59 °C for 1 min (×40); 98 °C for 10 min (×1); 50% ramp rate. Droplets were analyzed using a QX200 Droplet Reader (Bio-Rad) at FAM and HEX channels. Blank transfection served as a control to determine the threshold values to define NHEJ and HDR droplets. FAM++ fractions were identified as HDR population, FAM+ and HEX− fractions were identified as NHEJ population, and FAM+ and HEX+ fractions were identified as wild-type population^29^. Data acquisition and analysis were performed using QuantaSoft 1.6.6 (BioRad).

### Conversion of e*GFP* to *BFP*

U2OS.eGFP.PEST cells were transfected with RNP complex and ssODNs using the SE Cell Line 4D-Nucleofector kit (Lonza) following the DN-100 program. First, 30 pmol of Cas9 (or Cas9-adaptor) and 33 pmol of gRNA were mixed and incubated at RT for 5–10 min, then 60 pmol of ssODN was added, and the final mixture was incubated at RT for 15–30 min. After transfection, cells were suspended in the culture medium and transferred to a 96-well plate (20,000 cell per well). Seventy-two hours post transfection, the cells were fixed with a 4% paraformaldehyde solution, and the nuclei were stained with HCS NuclearMask Deep Red Stain (Invitrogen). The resulting fluorescence signals from eGFP, BFP, and nuclei were measured using an Operetta CLS High-Content Analysis System (PerkinElmer). Data acquisition and analysis was performed using Operetta Harmony 4.8 (PerkinElmer).

### *HiBiT* sequence knockin by nucleofection in INS-1E cells

INS-1E cells were transfected with Cas9 RNP and ssODN using the SF Cell Line 4D-Nucleofector kit (Lonza) following the pulse program of DE-130. For Cas9:ssODN conjugates, 20 pmol of Cas9-adaptor were premixed with 20 pmol of ssODN and incubated at RT for 15–30 min prior to RNP formation to ensure Cas9:ssODN conjugate formation. Then 20 pmol of gRNA was added, and the final mixture was incubated for 5–10 min at RT. In cases where Cas9 did not specifically bind ssODNs, the RNP was formed first because nonspecific Cas9-DNA interactions can hamper the RNP formation. After incubating Cas9 and gRNA at RT for 5–10 min, 20 pmol of ssODN were added to the mixture. Approximately 200,000 cells were transfected with the above mixtures in a well of the nucleofection kit, and cells were seeded in a well of a 24-well plate. Cells were incubated at 37 °C for 48 h, and the supernatant was taken to measure the amount of secreted HiBiT peptide using the Nano-Glo HiBiT Extracellular Detection System (Promega). The resulting luminescence signals were normalized based on the cell viability that was measured using the PrestoBlue Cell Viability Reagent (Thermo).

### *Il10* knockin by nucleofection in INS-1E cells

INS-1E cells were transfected with Cas9 RNP and ssODN using the SF Cell Line 4D-Nucleofector kit following the pulse program of DE-130. For Cas9:ssODN conjugates, 20 pmol of Cas9-adaptor were premixed with 12 pmol of ssODN and incubated at RT for 15–30 min prior to RNP formation to ensure Cas9:ssODN conjugate formation. Then 20 pmol of gRNA was added, and the final mixture was incubated for 5–10 min at RT. In cases where Cas9 did not specifically bind ssODNs, the RNP was formed first because nonspecific Cas9-DNA interactions can hamper the RNP formation. After incubating Cas9 and gRNA at RT for 5–10 min, 12 pmol of ssODN were added to the mixture. Approximately 200,000 cells were transfected with the above mixtures in a well of the nucleofection kit, and the cells were seeded in a well of a 24-well plate in 800 μL of the growth medium. The cells were incubated at 37 °C for 72 h, and the medium was taken to measure the amount of IL-10 accumulated for 72 h using the IL-10 Rat ELISA Kit (Invitrogen, catalog # BMS629). LPS was used at the concentration of 10 μg mL^−1^. The absorbance signals were recorded using SpectraMax M5 with SoftMax Pro 7.0 (Molecular Devices).

### Glucose-stimulated HiBiT secretion

INS-1E cells from the above genome editing experiments for *HiBiT* knockin were grown in a large scale. Then, cells were seeded in multiple wells of a 24-well plate at a density of 150,000 cells per well. The next day, cell were washed with and incubated in Krebs-Ringer bicarbonate (KRB) buffer (138 mM NaCl, 5.4 mM KCl, 5 mM NaHCO_3_, 2.6 mM MgCl_2_, 2.6 mM CaCl_2_, 10 mM HEPES, pH 7.4, 0.5% BSA) without glucose for 2 h. The cells in each well were subsequently incubated with different concentrations of glucose (from 2.80 to 16.8 mM) in the KRB buffer for 1 h. The supernatant was taken from each well to measure the amount of secreted HiBiT peptide using the Nano-Glo HiBiT Extracellular Detection System (Promega). The luminescence signal was recorded using Envision with PerkinElmer EnVision Manager 1.13 (PerkinElmer).

### Effect of secretagogues and diazoxide on the HiBiT secretion

INS-1E cells knocked in with *HiBiT* were enriched through sib-selection^34^, which gives consistent luminescence signal between wells of a 96-well plate. For the enrichment, the mixture of cells were plated in a 96-well plate at a density of 1000 cells per well. When the cells become confluent, the luminescence signal from the HiBiT secretion was recorded. Cells from the wells showing the highest signal were grown on a large scale, and the second round of enrichment was performed at a density of 100 cells per well. Finally enriched cells were used for the secretion assay. Those cells were seeded in a 96-well plate at a density of 40,000 cells per well. The next day, the cells were washed with PBS and were incubated in the KRB buffer containing 2.8 mM of glucose for 1 h. The cells in each well were subsequently incubated in the KRB containing different concentrations of glucose (2.80 mM, 9.80 mM, or 16.8 mM) and vehicle (water and DMSO), 40 mM of KCl, 50 *μ*M of 3-Isobutyl-1-methylxanthine (IBMX), 20 μM of phorbol 12-myristate 13-acetate (PMA), or 250 μM of diazoxide for 1 h. The supernatant was taken from each well to measure the amount of secreted HiBiT peptide. The luminescence signal was recorded by Envision with PerkinElmer EnVision Manager 1.13 (PerkinElmer).

### Glucose-stimulated IL-10 secretion

INS-1E cells knocked in with *Il10* were enriched through sib-selection^34^. The mixture of cells were plated in a 96-well plate at a density of 1,000 cells per well. When the cells become confluent, the IL-10 ELISA was performed using the IL-10 Rat ELISA Kit to identify wells with the highest IL-10 secretion. Cells from those wells were grown in a large scale, and the second round of enrichment was performed at a density of 100 cells per well using IL-10 ELISA as the readout. Finally enriched cells were seeded in a 96-well plate at a density of 50,000 cells per well. The next day, the cells were washed with PBS and were incubated in the KRB buffer containing 2.8 mM of glucose for 1 h. The cells in each well were subsequently incubated with different concentrations of glucose (2.80–16.8 mM) in the KRB buffer for 1 h. When investigating the effect of the compounds, the cells were incubated with glucose (2.80 mM or 16.8 mM) and DMSO, 50 μM of IBMX, or 250 μM of diazoxide for 1 h. The supernatant was taken to measure the amount of secreted IL-10 using the IL-10 Rat ELISA Kit.

### Correlation between insulin secretion and IL-10 secretion

INS-1E cells knocked in with *Il10* were seeded in a 96-well plate at a density of 50,000 cells per well. The next day, the cells were washed with PBS and were incubated in the KRB buffer containing 2.8 mM of glucose for 1 h. The cells in each well were subsequently incubated with varying concentrations of glucose (1.40, 2.80, 3.36, 4.20, 5.60, 9.80, 16.8 mM) in the KRB buffer for 1 h. The supernatant was taken to measure insulin content using the Rat insulin ELISA kit (APLCO, catalog # 80-INSRT-E01) and IL-10 content using the IL-10 Rat ELISA Kit.

### PCR to amplify the *Il10* knockin sequence

Genomic DNAs from the wild-type and edited INS-1E cells were extracted using a DNeasy Blood & Tissue Kit (Qiagen). PCR was performed using 50 ng of genomic DNA, 0.5 μM of forward primer, 0.5 μM of reverse primer, and Q5 Hot Start High-Fidelity 2x Master Mix (New England Biolabs) in a final volume of 25 μL. The PCR scheme is shown in Supplementary Fig. 15a. Primer sequences are listed in Supplementary Table 6. The HDR efficiency was measured by resolving the PCR product with 1% agarose gel containing SYBR gold (Invitrogen) and quantifying the band intensity with ImageJ 1.52a. The NHEJ efficiency was measured by T7EI assay using the same procedures as the above restriction endonuclease site knockin assay at *CXCR4*.

### Data analysis

All calculations and plotting were performed using Excel 2016 (Microsoft) and Prism 8 (GraphPad).

### Reporting summary

Further information on research design is available in the Nature Research Reporting Summary linked to this article.

## Supplementary information

Supplementary Information

Reporting Summary

## Data Availability

The data generated in this study are provided in the manuscript and the supplementary information, and are available from the corresponding author upon reasonable request. Plasmids from Addgene (#98158 [https://www.addgene.org/98158], and #70219 [https://www.addgene.org/70219]) were used in this study. Structural information from PDB (ID: 5F9R [https://www.rcsb.org/structure/5F9R], 4CMP [https://www.rcsb.org/structure/4CMP], and 4ZT0 [https://www.rcsb.org/structure/4ZT0]) was used in this study. Source data are provided with this paper.

## Source data

Source Data
