# Supplementary material for: Engineering designer beta cells with a CRISPR-Cas9 conjugation platform
Source: Nat Commun. 2020 Aug 13;11:4043. doi: 10.1038/s41467-020-17725-0 (PMC7426819; doi:10.1038/s41467-020-17725-0)
Supplement: Supplementary file 1 — Supplementary Information [file 41467_2020_17725_MOESM1_ESM.pdf]

Supplementary Information

Engineering designer beta cells with a CRISPR-Cas9 conjugation platform

Lim et. al.

This file contains:

Supplementary Tables and Figures 2−25

Supplementary References 26

1. Supplementary Tables and Figures

Supplementary Table 1. Primer sequences for mutagenesis.

Mutation site	Primer sequence
C80S	F: CGTATTAGCTATCTACAGGAGATTTTTTCAAATGAG<br>R: GTAGATAGCTAATACGATTCTTCCGACGTG
C574S	F: AAAAATAGAAAGCTTTGATAGTGTTGAAATTTC<br>R: TTGAAATAATCTTCTTTTAATTGC
M1C	F: GAGGAAGGTGTGCGATAAGAAATACTCAATAGG<br>R: TTCTTCTTGGGCATAAAC
S204C	F: TATTAACGCATGCGGAGTAGATGC<br>R: GGGTTTTCTTCAAATAATTGATTG
E532C	F: GTTACTTGCGGAATGCGAAAACCAGCATTTC<br>R: CGCATTCCGCAAGTAACATATTTGACCTTTGTC
K558C	F: AACAAATCGATGCGTAACCGTTAAGCAATTAAAAG<br>R: TTGAAGAGTAAATCAACAATG
Q826C	F: GTATGTGGACTGCGAATTAGATATTAATCGTTTAAG<br>R: ATGTCTCTTCCATTTTGG
E945C	F: TAAATACGATTGCAATGATAAACTTATTCGAG<br>R: GTATTCATGCGACTATCC
E1026C	F: TGCTAAGTCTTGCCAAGAAATAGGC<br>R: ATCATTTTACGAACATCATAAAC
N1054C	F: TACACTTGCATGCGGAGAGATTCGC<br>R: ATTTCTGTTTTGAAGAAGTTC
E1068C	F: CTAATGGGTGCACTGGAGAAATTGTCTGGG<br>R: CTCCAGTGCACCCATTAGTTTCGATTAGAGGG
S1116C	F: AAAAAGAAATTGCGACAAGCTTATTGCTC<br>R: GGTAAAATTGACTCCTTGG
K1153C	F: AAAAGGGTGCTCGAAGAAGTTAAAATCCGTTAAAGAG<br>R: CTTCGAGCACCCTTTTTCCACCTTAGCAAC
E1207C	F: TTTTGAGTTATGCAACGGTCGTAAACG<br>R: AGACTATATTTAGGTAGTTTAATG

Supplementary Table 2. Primer sequences for gRNA synthesis.

Primer name	Primer sequence
Universal reverse	AAAAGCACCGACTCGGTGCCACTTTTTCAAGTTGATAACGGACTAGCCTTATTTTAACTTGCTATTTCTAGCTCTAAAAC
GAPDH 1 forward	TAATACGACTCACTATAGGTCCAGGGGTCTTACTCCTGTTTTAGAGCTAGAAAT
GAPDH 2 forward	TAATACGACTCACTATAGCCTCCAAGGAGTAAGACCCCGTTTTAGAGCTAGAAAT
PPIB forward	TAATACGACTCACTATAGCGCCAAGGAGTAGGGCACAGTTTTAGAGCTAGAAAT
CFL1 forward	TAATACGACTCACTATAGGGCCAGAAGGGGCTCACAAGTTTTAGAGCTAGAAAT
CXCR4 forward	TAATACGACTCACTATAGAAGCGTGATGACAAAGAGGGTTTTAGAGCTAGA AAT
RBM20 1 forward	TAATACGACTCACTATAGGGACCTCGGGGAGAGTGACGTTTTAGAGCTAGAAAT
RBM20 2 forward	TAATACGACTCACTATAGGGGAGAGTGACCGGCTCACGTTTTAGAGCTAGAAAT
Ins1 1a forward	TAATACGACTCACTATAGCCCAAGTCCCGTCGTGAAGGTTTTAGAGCTAGAAAT
Ins1 1b forward	TAATACGACTCACTATAGCTCCAGTTGTGGCACTTGCGTTTTAGAGCTAGAAAT
Ins1 2a forward	TAATACGACTCACTATAGGGTGGAGGCCCGGAGGCCGTTTTAGAGCTAGAAAT
Ins1 2b forward	TAATACGACTCACTATAGGGTGGAGGCCCGGAGGCCGGTTTTAGAGCTAGAAAT
Ins1 2c forward	TAATACGACTCACTATAGTCTGAAGATCCCCGGCCTCGTTTTAGAGCTAGAAAT
Ins1 2d forward	TAATACGACTCACTATAGTGGGTGGAGGCCCGGAGGCGTTTTAGAGCTAGAAAT
Ins1 2e forward	TAATACGACTCACTATAG CTGAAGATCCCCGGCCTCCGTTTTAGAGCTAGAAAT
Ins1 3a forward	TAATACGACTCACTATAGACAATGCCACGCTTCTGCCGTTTTAGAGCTAGAAAT
Ins1 3b forward	TAATACGACTCACTATAGCTTCAGACCTTGGCACTGGGTTTTAGAGCTAGAAAT
eGFP (Suppl. Fig. 4) matched forward	TAATACGACTCACTATAGGGCACGGGCAGCTTGCCGGGTTTTAGAGCTAGAAAT
eGFP (Suppl. Fig. 4) mismatched 1 forward	TAATACGACTCACTATAGGGCACGGGCAGCTTGCCGCGTTTTAGAGCTAGAAAT
eGFP (Suppl. Fig. 4) mismatched 2 forward	TAATACGACTCACTATAGGGCACGGGCAGCTTGCCCGGTTTTAGAGCTAGAAAT
eGFP (Suppl. Fig. 4) mismatched 5 forward	TAATACGACTCACTATAGGGCACGGGCAGCTTCCCGGGTTTTAGAGCTAGAAAT
eGFP (Suppl. Fig. 11) forward	TAATACGACTCACTATAGTCGTGACCACCCTGACCTAGTTTTAGAGCTAGAAAT

Supplementary Table 3. Sequences of ssODNs (≤ 200 nt).

ssODN name	Assay	ssODN sequence
GAPDH adaptor	NanoLuc luciferase complementation	TCTTCTAGGTATGACAACGAATTTGGCTACAGCAACAGGGTGGTGGAC CTCATGGCCCACATGGCCTCCAAGGAGGTGAGCGGCTGGCGGCTGTT CAAGAAGATTAGCTAAGACCCCTGGACCACCAGCCCCAGCAAGAGCA CAAGAGGAAGAGAGAGACCCTCACTGCTGGGGAGTCCCTGCGACGAT GAGAGTGAAGC
GAPDH adaptor-free	NanoLuc luciferase complementation	TCTTCTAGGTATGACAACGAATTTGGCTACAGCAACAGGGTGGTGGAC CTCATGGCCCACATGGCCTCCAAGGAGGTGAGCGGCTGGCGGCTGTT CAAGAAGATTAGCTAAGACCCCTGGACCACCAGCCCCAGCAAGAGCA CAAGAGGAAGAGAGAGACCCTCACTGCTGGGGAGTCCCTGC
GAPDH 15-nt adaptor	NanoLuc luciferase complementation	TCTTCTAGGTATGACAACGAATTTGGCTACAGCAACAGGGTGGTGGAC CTCATGGCCCACATGGCCTCCAAGGAGGTGAGCGGCTGGCGGCTGTT CAAGAAGATTAGCTAAGACCCCTGGACCACCAGCCCCAGCAAGAGCA CAAGAGGAAGAGAGAGACCCTCACTGCTGGGGAGTCCCTGCGACGAT GAGAGTGAA
GAPDH 13-nt adaptor	NanoLuc luciferase complementation	TCTTCTAGGTATGACAACGAATTTGGCTACAGCAACAGGGTGGTGGAC CTCATGGCCCACATGGCCTCCAAGGAGGTGAGCGGCTGGCGGCTGTT CAAGAAGATTAGCTAAGACCCCTGGACCACCAGCCCCAGCAAGAGCA CAAGAGGAAGAGAGAGACCCTCACTGCTGGGGAGTCCCTGCGACGAT GAGAGTG
PPIB adaptor	NanoLuc luciferase complementation	CAGCTCAGAGCCCTGTGGCGGACTACAGGGCCTGCACAGACGGTCAC TCAAAGAAAGATGTCCCTGTGCCCTAGCTAATCTTCTTGAACAGCCGC CAGCCGCTCACCTCCTTGGCGATGGCAAAGGGCTTCTCCACCTCGAT CTTGCCGCAGTCTGCGATGATCACATCCTTCAGGGGTGACGATGAGA GTGAAGC
PPIB adaptor-free	NanoLuc luciferase complementation	CAGCTCAGAGCCCTGTGGCGGACTACAGGGCCTGCACAGACGGTCAC TCAAAGAAAGATGTCCCTGTGCCCTAGCTAATCTTCTTGAACAGCCGC CAGCCGCTCACCTCCTTGGCGATGGCAAAGGGCTTCTCCACCTCGAT CTTGCCGCAGTCTGCGATGATCACATCCTTCAGGGGT
CFL1 adaptor	NanoLuc luciferase complementation	GAGGTCAAGGACCGCTGCACCCTGGCAGAGAAGCTGGGGGGCAGTG CCGTCATCTCCCTGGAGGGCAAGCCTTTGGTGAGCGGCTGGCGGCTG TTCAAGAAGATTAGCTGAGCCCCTTCTGGCCCCCTGCCTGGAGCATCT GGCAGCCCCACACCTGCCCTTGGGGGTTGCAGGCTGCCCCCTGACGA TGAGAGTGAAGC
CFL1 adaptor-free	NanoLuc luciferase complementation	GAGGTCAAGGACCGCTGCACCCTGGCAGAGAAGCTGGGGGGCAGTG CCGTCATCTCCCTGGAGGGCAAGCCTTTGGTGAGCGGCTGGCGGCTG TTCAAGAAGATTAGCTGAGCCCCTTCTGGCCCCCTGCCTGGAGCATCT GGCAGCCCCACACCTGCCCTTGGGGGTTGCAGGCTGCCCCCT
GAPDH adaptor	GFP complementation	GACAACGAATTTGGCTACAGCAACAGGGTGGTGGACCTCATGGCCCA CATGGCCTCCAAGGAGGGTGGCGGCCGTGACCACATGGTCCTTCATG AGTATGTAAATGCTGCTGGGATTACATAAGACCCCTGGACCACCAGCC CCAGCAAGAGCACAAGAGGAAGAGAGAGACCCTCACTGCTGGACGAT GAGAGTGAAGC
GAPDH adaptor-free	GFP complementation	GACAACGAATTTGGCTACAGCAACAGGGTGGTGGACCTCATGGCCCA CATGGCCTCCAAGGAGGGTGGCGGCCGTGACCACATGGTCCTTCATG AGTATGTAAATGCTGCTGGGATTACATAAGACCCCTGGACCACCAGCC CCAGCAAGAGCACAAGAGGAAGAGAGAGACCCTCACTGCTG
CXCR4	Restriction site insertion	TAGATGACATGGACTGCCTTGCATAGGAAGTTCCCAAAGTACCAGTTT GCCACGGCATCAACTGCCCAGAAGGGAAGCGTGATGGCATGCAAGCT TTCGGCCACTGACAGGTGCAGCCTGTACTTGTCCGTCATGCTTCTCAG TTTCTTCTGGTAACCCATGACCAGGATGACCAATCCAGACGATGAGAG TGAAGC

RBM20 #1 adaptor	Droplet digital PCR	GTGGGAAGAGCTGCAGGAGGTGAAGCTGGGAGTGTGGGACCTCGGTGAGAGTGACCGGCTCACCGGACTACTAGACCGCGGCCTTTCTGGGCCATATCTGTGAGGGAGCCAAGGAGCAGGGACGATGAGAGTGAAGC
RBM20 #2 adaptor	Droplet digital PCR	ACAGATATGGCCCAGAAAGGCCGCGGTCTAGTAGTCCGGTGAGCCGGTCACTGTCCCCGAGGTCCCACACACCCAGCGACGATGAGAGTGAAGC
eGFP #1	Converting *eGFP* to *BFP*	CTGAAGTTCATCTGCACCACCGGCAAGCTGCCCGTGCCCTGGCCCACCCTCGTGACCACCCTGAGCCACGGGGTGCAGTGCTTCAGCCGCTACCCCGACCACATGAAGCAGCACGACTTCTTCAAGTCCGCCGACGATGAGAGTGAAGC
eGFP #2	Converting *eGFP* to *BFP*	TACGGCAAGCTGACCCTGAAGTTCATCTGCACCACCGGCAAGCTGCCCGTGCCCTGGCCCACCCTCGTGACCACCCTGAGCCACGGCGTGCAGTGCTTCAGCCGCTACCCCGACCACATGAAGCAGCACGACTTCTTCAAGTCCGCCATGCCCGAAGGCTACGACGATGAGAGTGAAGC
Ins1 site 1 adaptor-free	NanoLuc luciferase complementation	GGAGGCTCTGTACCTGGTGTGTGGGGAACGTGGTTTCTTCTACACACCCAAGTCCCGTCGTGAAGTGGAGAAGCGTGTGAGCGGCTGGCGGCTGTTCAAGAAGATTAGCAAGCGTGACCCGCAAGTGCCACAACTGGAGCTGGGTGGAGGCCCGGAGGCCGGGGATCTTCAGACCTTGGCACTGG
Ins1 site 2 adaptor-free	NanoLuc luciferase complementation	ACACCCAAGTCCCGTCGTGAAGTGGAGGACCCGCAAGTGCCACAACTGGAGCTGGGTGGAGGCCCGGAGAAGCGTGTGAGCGGCTGGCGGCTGTTCAAGAAGATTAGCAAGCGTGCCGGGGATCTTCAGACCTTGGCACTGGAGGTTGCCCGGCAGAAGCGTGGCATTGTGGATCAGTGCTGC
Ins1 site 2 adaptor	NanoLuc luciferase complementation	ACACCCAAGTCCCGTCGTGAAGTGGAGGACCCGCAAGTGCCACAACTGGAGCTGGGTGGAGGCCCGGAGAAGCGTGTGAGCGGCTGGCGGCTGTTCAAGAAGATTAGCAAGCGTGCCGGGGATCTTCAGACCTTGGCACTGGAGGTTGCCCGGCAGAAGCGTGGCATTGTGGATCAGTGCTGCGACGATGAGAGTGAAGC
Ins1 site 3 adaptor-free	NanoLuc luciferase complementation	GCAAGTGCCACAACTGGAGCTGGGTGGAGGCCCGGAGGCCGGGGATCTTCAGACCTTGGCACTGGAGGTTAAGCGTGTGAGCGGCTGGCGGCTGTTCAAGAAGATTAGCAAGCGTGCCCGGCAGAAGCGTGGCATTGTGGATCAGTGCTGCACCAGCATCTGCTCCCTCTACCAACTGGAGAACT

Supplementary Table 4. Sequences of gBlocks DNAs and primers for generating ssODNs for *IL-10* knock-in.

DNA name	DNA sequence
gBlocks *Ins1-Il10* adaptor	TAATACGACTCACTATAGCTTCACTCTCATCGTCGGCTTTATTCATTGCAGAGGGGTGGGCGGG GAGTGGTGGACTCAGTTGCAGTAGTTCTCCAGTTGGTAGAGGGAGCAGATGCTGGTGCAGCA CTGATCCACAATGCCACGCTTCTGCCGGGCAACCTCCAGTGCCAAGGTCTGAAGATCCCCGG CACGCTTATTTTTCATTTTGAGTGTCACGTAGGCTTCTATGCAGTTGATGAAGATGTCAAACTCA TTCATGGCCTTGTAGACACCTTTGTCTTGGAGCTTATTAAAATCATTCTTCACCTGCTCCACTGC CTTGCTTTTATTCTCACAGGGGAGAAATCGATGACAGCGTCGCAGCTGTATCCAGAGGGTCTT CAGCTTCTCTCCCAGGGAATTCAAATGCTCCTTGATTTCTGGGCCATGGTTCTCTGCCTGGGG CATCACTTCTACCAGGTAAAACTTGATCATTTCTGACAAGGCTTGGCAACCCAAGTAACCCTTA AAGTCCTGCAGTAAGGAATCTGTCAGCAGTATGTTGTCCAGCTGGTCCTTCTTTTGAAAGAAAG TCTTCACTTGACTGAAGGCAGCCCTCAGCTCTCGGAGCATGTGGGTCTGGCTGACTGGGAAGT GGGTGCAGTTATTGTCACCCCGGATGGAATGGCCTTTGCTACGCTTCTCCGGGCCTCCACCCA GCTCCAGTTGTGGCACTTGCGGGTCCTCCACTTCACGACGGGACTTGGGTGTGTAGAAGAAAC CACGTTCCCCACACACCAGGTACAGAGCCTCCACCAGGTGAGGACCACAAAGGTGCTGTTTGA CAAAAGC
gBlocks *Ins1-Il10* adaptor-free	TAATACGACTCACTATAGGCTTTATTCATTGCAGAGGGGTGGGCGGGGAGTGGTGGACTCAGT TGCAGTAGTTCTCCAGTTGGTAGAGGGAGCAGATGCTGGTGCAGCACTGATCCACAATGCCAC GCTTCTGCCGGGCAACCTCCAGTGCCAAGGTCTGAAGATCCCCGGCACGCTTATTTTTCATTTT GAGTGTCACGTAGGCTTCTATGCAGTTGATGAAGATGTCAAACTCATTCATGGCCTTGTAGACA CCTTTGTCTTGGAGCTTATTAAAATCATTCTTCACCTGCTCCACTGCCTTGCTTTTATTCTCACA GGGGAGAAATCGATGACAGCGTCGCAGCTGTATCCAGAGGGTCTTCAGCTTCTCTCCCAGGG AATTCAAATGCTCCTTGATTTCTGGGCCATGGTTCTCTGCCTGGGGCATCACTTCTACCAGGTA AAACTTGATCATTTCTGACAAGGCTTGGCAACCCAAGTAACCCTTAAAGTCCTGCAGTAAGGAA TCTGTCAGCAGTATGTTGTCCAGCTGGTCCTTCTTTTGAAAGAAAGTCTTCACTTGACTGAAGG CAGCCCTCAGCTCTCGGAGCATGTGGGTCTGGCTGACTGGGAAGTGGGTGCAGTTATTGTCA CCCCGGATGGAATGGCCTTTGCTACGCTTCTCCGGGCCTCCACCCAGCTCCAGTTGTGGCACT TGCGGGTCCTCCACTTCACGACGGGACTTGGGTGTGTAGAAGAAACCACGTTCCCCACACACC AGGTACAGAGCCTCCACCAGGTGAGGACCACAAAGGTGCTGTTTGACAAAAGC
Ins1 forward adaptor	TAATACGACTCACTATAGCTTCACTCTCATCG
Ins1 forward adaptor-free	TAATACGACTCACTATAGGCTTTATTCATTGCAGAGGGGTGG
Ins1 reverse universal	GCTTTTGTCAAACAGCACCTT

Supplementary Table 5. Sequences of long ssODNs for *IL-10* knock-in.

ssODN name	Assay	ssODN sequence
Ins1-Il10 adaptor	IL-10 ELISA	GCTTTTGTCAAACAGCACCTTTGTGGTCCTCACCTGGTGGAGGCTCTGTAC<br>CTGGTGTGTGGGGAACGTGGTTTCTTCTACACACCCAAGTCCCGTCGTGAA<br>GTGGAGGACCCGCAAGTGCCACAACTGGAGCTGGGTGGAGGCCCGGAGA<br>AGCGTAGCAAAGGCCATTCCATCCGGGGTGACAATAACTGCACCCACTTCC<br>CAGTCAGCCAGACCCACATGCTCCGAGAGCTGAGGGCTGCCTTCAGTCAA<br>GTGAAGACTTTCTTTCAAAAGAAGGACCAGCTGGACAACATACTGCTGACA<br>GATTCCTTACTGCAGGACTTTAAGGGTTACTTGGGTTGCCAAGCCTTGTCA<br>GAAATGATCAAGTTTTACCTGGTAGAAGTGATGCCCCAGGCAGAGAACCAT<br>GGCCCAGAAATCAAGGAGCATTTGAATTCCCTGGGAGAGAAGCTGAAGAC<br>CCTCTGGATACAGCTGCGACGCTGTCATCGATTTCTCCCCTGTGAGAATAA<br>AAGCAAGGCAGTGGAGCAGGTGAAGAATGATTTTAATAAGCTCCAAGACAA<br>AGGTGTCTACAAGGCCATGAATGAGTTTGACATCTTCATCAACTGCATAGAA<br>GCCTACGTGACACTCAAAATGAAAAATAAGCGTGCCGGGGATCTTCAGACC<br>TTGGCACTGGAGGTTGCCCGGCAGAAGCGTGGCATTGTGGATCAGTGCTG<br>CACCAGCATCTGCTCCCTCTACCAACTGGAGAACTACTGCAACTGAGTCCA<br>CCACTCCCCGCCCACCCCTCTGCAATGAATAAAGCCGACGATGAGAGTGA<br>AGC
Ins1-Il10 adaptor-free	IL-10 ELISA	GCTTTTGTCAAACAGCACCTTTGTGGTCCTCACCTGGTGGAGGCTCTGTAC<br>CTGGTGTGTGGGGAACGTGGTTTCTTCTACACACCCAAGTCCCGTCGTGAA<br>GTGGAGGACCCGCAAGTGCCACAACTGGAGCTGGGTGGAGGCCCGGAGA<br>AGCGTAGCAAAGGCCATTCCATCCGGGGTGACAATAACTGCACCCACTTCC<br>CAGTCAGCCAGACCCACATGCTCCGAGAGCTGAGGGCTGCCTTCAGTCAA<br>GTGAAGACTTTCTTTCAAAAGAAGGACCAGCTGGACAACATACTGCTGACA<br>GATTCCTTACTGCAGGACTTTAAGGGTTACTTGGGTTGCCAAGCCTTGTCA<br>GAAATGATCAAGTTTTACCTGGTAGAAGTGATGCCCCAGGCAGAGAACCAT<br>GGCCCAGAAATCAAGGAGCATTTGAATTCCCTGGGAGAGAAGCTGAAGAC<br>CCTCTGGATACAGCTGCGACGCTGTCATCGATTTCTCCCCTGTGAGAATAA<br>AAGCAAGGCAGTGGAGCAGGTGAAGAATGATTTTAATAAGCTCCAAGACAA<br>AGGTGTCTACAAGGCCATGAATGAGTTTGACATCTTCATCAACTGCATAGAA<br>GCCTACGTGACACTCAAAATGAAAAATAAGCGTGCCGGGGATCTTCAGACC<br>TTGGCACTGGAGGTTGCCCGGCAGAAGCGTGGCATTGTGGATCAGTGCTG<br>CACCAGCATCTGCTCCCTCTACCAACTGGAGAACTACTGCAACTGAGTCCA<br>CCACTCCCCGCCCACCCCTCTGCAATGAATAAAGCC

Supplementary Table 6. Primer and probe sequences used for droplet digital PCR and genotyping experiments.

DNA name	Sequence
RBM20 Forward	CTGTGTGTGGGTGGGGT
RBM20 Reverse	AGGAGGTGAAGCTGGGAG
RBM20 reference probe	FAM-TGGGAGGTG/ZEN/TGAAGATTCTAAATC-IABkFQ
RBM20 HDR probe	FAM-CCGCGGTCT/ZEN/AGTAGTCC-IABkFQ
RBM20 NHEJ probe	HEX-AGAGTGACC/ZEN/GGCTCAC-IABkFQ
RBM20 dark probe	CCGCGGTCTCGTAGTCC-phosphate
CXCR4 Forward	GACTTTGAAACCCTCAGCGTC
CXCR4 Reverse	AGTCATTGGGGTAGAAGCGG
Ins1 Forward 1	CCCGGAGAAGCGTAGCAAAG
Ins1 Forward 2	GTCCAATGAGCGCTTTCTGC
Ins1 Reverse 1	AAAGATTCCCGTTCACACAATCC

Supplementary Table 7. Absolute genome editing efficiencies.

Assay	Editing efficiency (%)	Figures
GFP11 knock-in	**Wild type (HDR, adaptor ssODN):** 0.359, 0.309, 0.348<br>**532 (HDR, adaptor ssODN):** 1.64, 0.780, 1.32<br>**Wild type (HDR, no_adaptor ssODN):** 0.456, 0.460, 0.494<br>**532 (HDR, no_adaptor ssODN):** 0.493, 0.427, 0.459	3b / Supplementary 7d
CXCR4 12-base exchange	**Wild type (NHEJ):** 25.4, 29.9, 21.4, **Wild type (HDR):** 2.88, 2.32, 1.55<br>**532 (NHEJ):** 36.5, 30.6, 33.0, **532 (HDR):** 8.46, 6.40, 5.64<br>**945 (NHEJ):** 32.6, 38.8, 28.2, **945 (HDR):** 6.91, 10.1, 5.62	3c
RBM20 2-base exchange	**Wild type (NHEJ):** 2.11, 0.828, 0.882; **Wild type (HDR):** 0.0631, 0.0126, 0.0141<br>**1 (NHEJ):** 3.34, 3.14, 2.19; **1 (HDR):** 0.140, 0.165, 0.0929	3d
RBM20 2-base exchange	**Wild type (NHEJ)**: 1.38, 1.17, 1.30; **Wild type (HDR)**: 0.0151, 0.0194, 0.0184<br>**532 (NHEJ):** 3.65, 4.55, 3.60; **532 (HDR):** 0.206, 0.215, 0.172	3d
RBM20 2-base exchange	**Wild type (NHEJ):** 0.716, 1.05, 0.871; **Wild type (HDR):** 0.0151, 0.0103, 0.0135<br>**945 (NHEJ):** 1.13, 3.05, 1.17; **945 (HDR):** 0.0412, 0.142, 0.0471	3d
RBM20 2-base exchange	**Wild type (NHEJ):** 1.38, 0.871, 0.828; **Wild type (HDR):** 0.0151, 0.0135, 0.0126<br>**1026 (NHEJ):** 1.80, 2.43, 3.57; **1026 (HDR):** 0.0749, 0.0911, 0.170	3d
RBM20 2-base exchange	**Wild type (NHEJ):** 1.30, 1.38, 0.676 ; **Wild type (HDR):** 0.0184, 0.0151, 0.00963<br>**1207 (NHEJ):** 1.17, 1.06, 1.30; **1207 (HDR):** 0.0515, 0.0564, 0.0466	3d
RBM20 3-base exchange	**Wild type (NHEJ):** 2.37, 2.57; **Wild type (HDR):** 0.0623, 0.0688<br>**532 (NHEJ):** 4.91, 4.65; **532 (HDR):** 0.243, 0.219	Supplementary 10c
RBM20 3-base exchange	**Wild type (NHEJ):** 2.57, 1.79; **Wild type (HDR):** 0.0688, 0.0524<br>**945 (NHEJ):** 4.32, 4.77; **945 (HDR):** 0.208, 0.228	Supplementary 10c
RBM20 2-base exchange	**Wild type (NHEJ):** 0.528, 0.463, 1.60; **Wild type (HDR):** 0.00673, 0.00742, 0.0348<br>**532 (NHEJ):** 1.05, 0.493, 1.94; **532 (HDR):** 0.0241, 0.0109, 0.0503<br>**945 (NHEJ):** 0.700, 0.378, 1.62; **945 (HDR):** 0.0189, 0.0143, 0.0465<br>**532/945 (NHEJ):** 1.65, 0.614; 1.68; **532/945 (HDR):** 0.0583, 0.0271, 0.0691	4c
RBM20 2-base exchange	**Wild type (NHEJ):** 0.415, 0.863, 0.353; **Wild type (HDR):** 0.00836, 0.0197, 0.00413<br>**532 (NHEJ):** 0.444, 0.837, 0.485; **532 (HDR):** 0.0136, 0.0364, 0.0131<br>**1207 (NHEJ):** 0.283, 0.437, 0.290; **1207 (HDR):** 0.00995, 0.0204, 0.0161<br>**532/1207 (NHEJ):** 0.282, 0.211, 0.286; **532/1207 (HDR):** 0.0187, 0.0151, 0.0201	4d
eGFP to *BFP* 3-base exchange ssODN #1	**Wild type (NHEJ):** 96.3, 95.7; **Wild type (HDR):** 3.01, 3.81<br>**945 (NHEJ):** 94.0, 94.6; **945 (HDR):** 5.26, 5.01	Supplementary 11b
eGFP to *BFP* 2-base exchange ssODN #2	**Wild type (NHEJ):** 73.8, 72.6; **Wild type (HDR):** 25.5, 27.0<br>**945 (NHEJ):** 67.5, 66.1; **945 (HDR):** 31.9, 33.2	Supplementary 11b

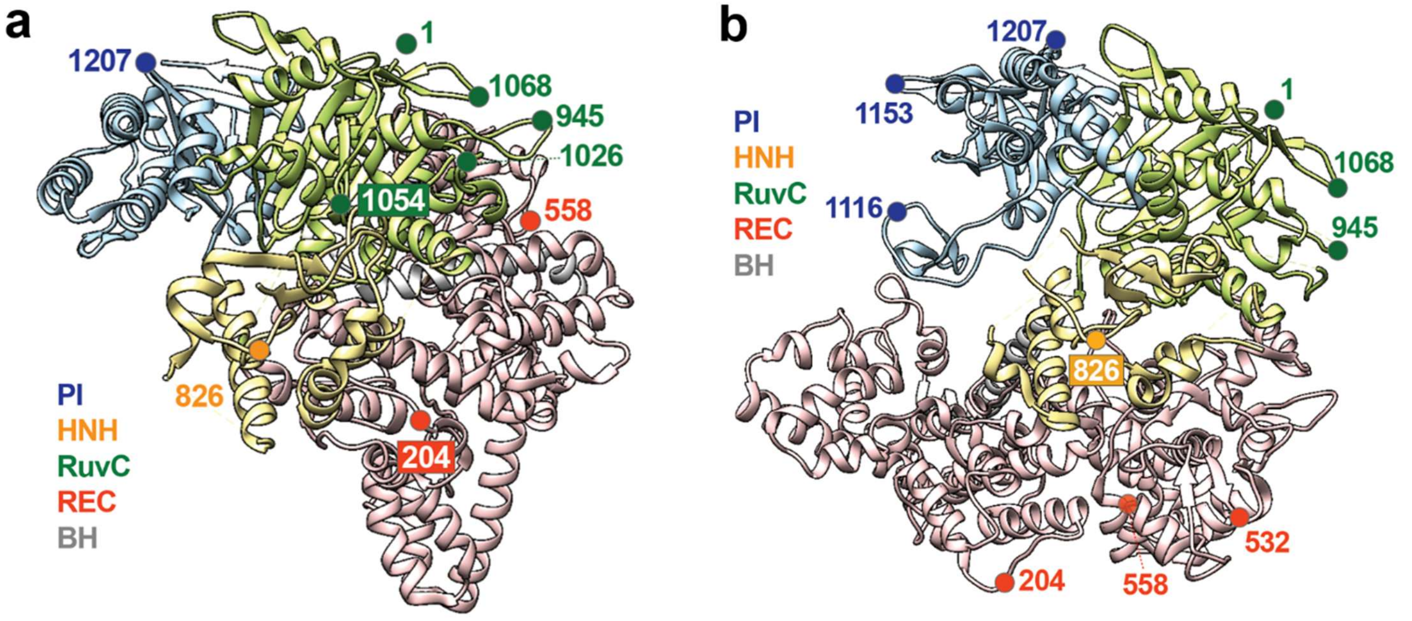

Supplementary Figure 1. Selection of Cas9 labeling sites based on crystal structures. (a) Structure of apo-Cas9 (PDB ID: 4CMP).[1] Four residues (1, 532, 1116, 1153) are not assigned at the structure possibly due to the high flexibility. We assumed that those sites are surface-exposed based on the nucleic-acid-bound structures and/or high flexibility of the loops they belong to. (b) Structure of gRNA-bound Cas9 (PDB ID: 4ZT0).[2] Only residue 558 is projected toward the interior of the protein, indicating that labeling at this site can inhibit the formation of the correct ribonucleoprotein (RNP) structure. Cas9 exhibits a large conformational change, especially at the recognition (REC) lobe, upon gRNA binding (residues 204, 532, 558). Protospacer adjacent motif-interacting (PI) domain is in blue, HNH domain is in yellow, RuvC domain is in green, recognition (REC) lobe is in red, and bridge helix (BH) is in gray.

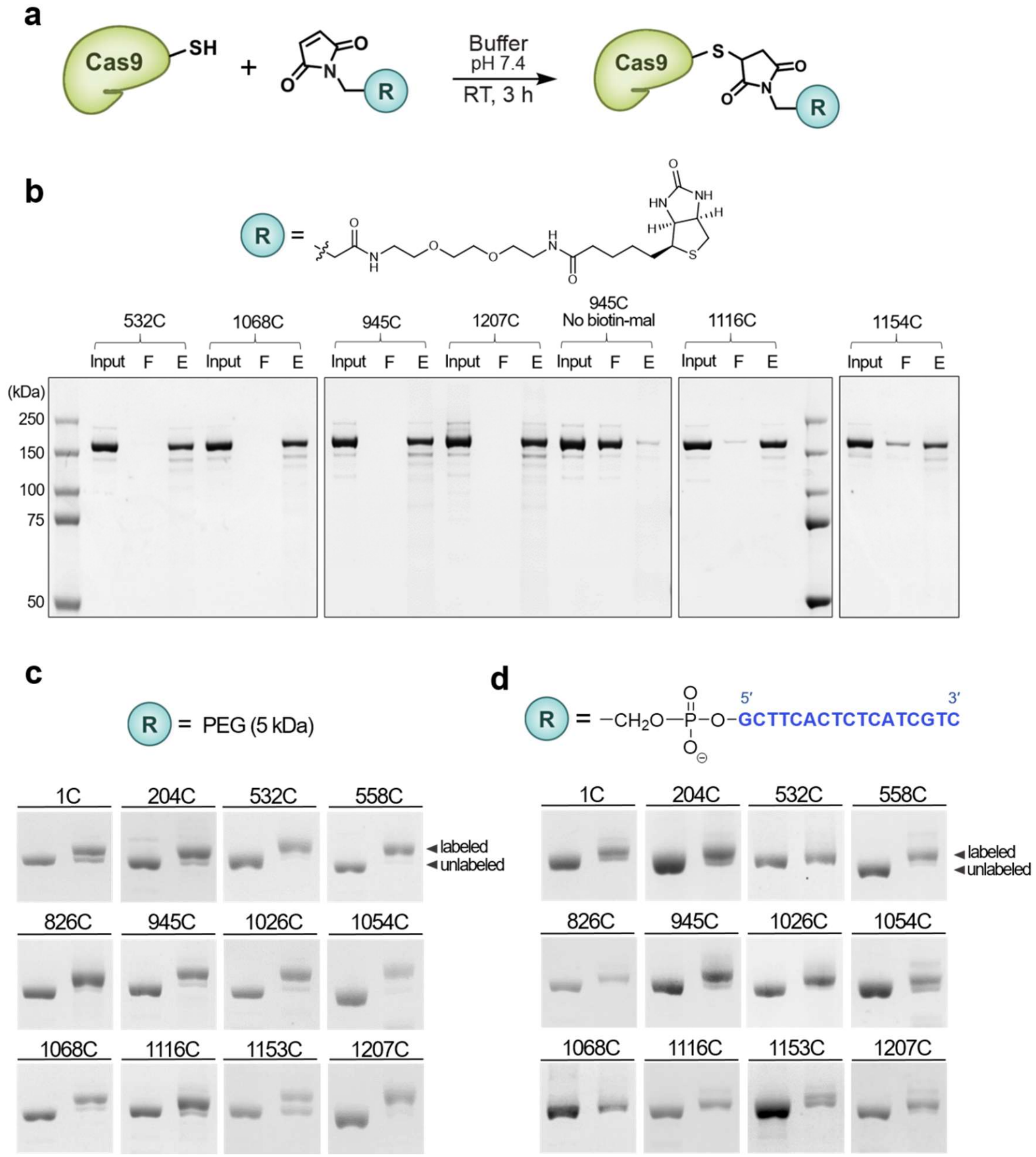

Supplementary Figure 2. Chemical labeling of Cas9. (a) Schematic of the site-specific labeling of Cas9 single-cysteine mutants by thiol-maleimide conjugation. (b) Biotin-maleimide was reacted with a cysteine on Cas9. The reaction mixture was subjected to pull-down by streptavidin beads to separate between unlabeled (flow-through, F) and biotinylated (eluate, E) Cas9. Each fraction was analyzed by SDS-PAGE followed by Coomassie staining. The images show representative results from two independent experiments (945C, 1154C) or results from single experiments (532C, 1068C, 1207C, 945C No biotin-mal, 1116C). (c) PEG-maleimide was reacted with a cysteine on Cas9. The images show representative results from three independent experiments (1207C), representative results from two independent experiments (1C, 532C, 1068C), or results from single experiments (204C, 558C, 826C, 945C, 1026C, 1054C, 1116C, 1153C). (d) The adaptor oligonucleotide with a 5′-maleimide group was reacted with a cysteine on Cas9. The images show representative results from five independent experiments (532C, 945C), representative results from three independent experiments (1026C, 1068C, 1207C), representative results from two independent experiments (1C, 204C, 558C, 826C, 1054C, 1116C), or the result from a single experiment (1153C). The degree of labeling was monitored through SDS-PAGE followed by Coomassie staining for PEG and DNA labeling. Full-gel images are provided as a Source Data file.

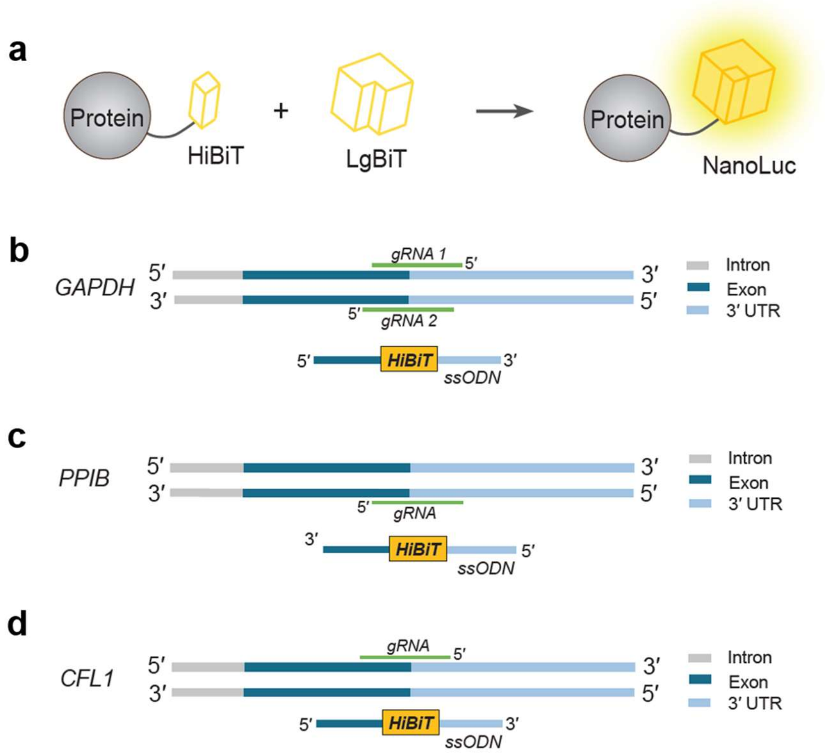

Supplementary Figure 3. Schematic of the HiBiT assay to check the HDR-mediated knock-in of the 33-nt DNA fragment. (a) *HiBiT* sequence knock-in right before the stop codon of the gene of interest results in the expression of a fusion protein having a C-terminal HiBiT tag, which is a small fragment of the NanoLuc luciferase. When an excess amount of the other fragment of NanoLuc (LgBiT) is supplied, a fully functional NanoLuc is reconstituted. The resulting luminescence signal is proportional to the HDR efficiency. (b) Design strategy for *HiBiT* knock-in at the *GAPDH* locus. gRNA 1 was used for genome editing in Fig. 2, and gRNA 2 was used in Fig. 3a. (c-d) Design strategy for *HiBiT* knock-in at (c) the *PPIB* locus and (d) the *CFL1* locus.

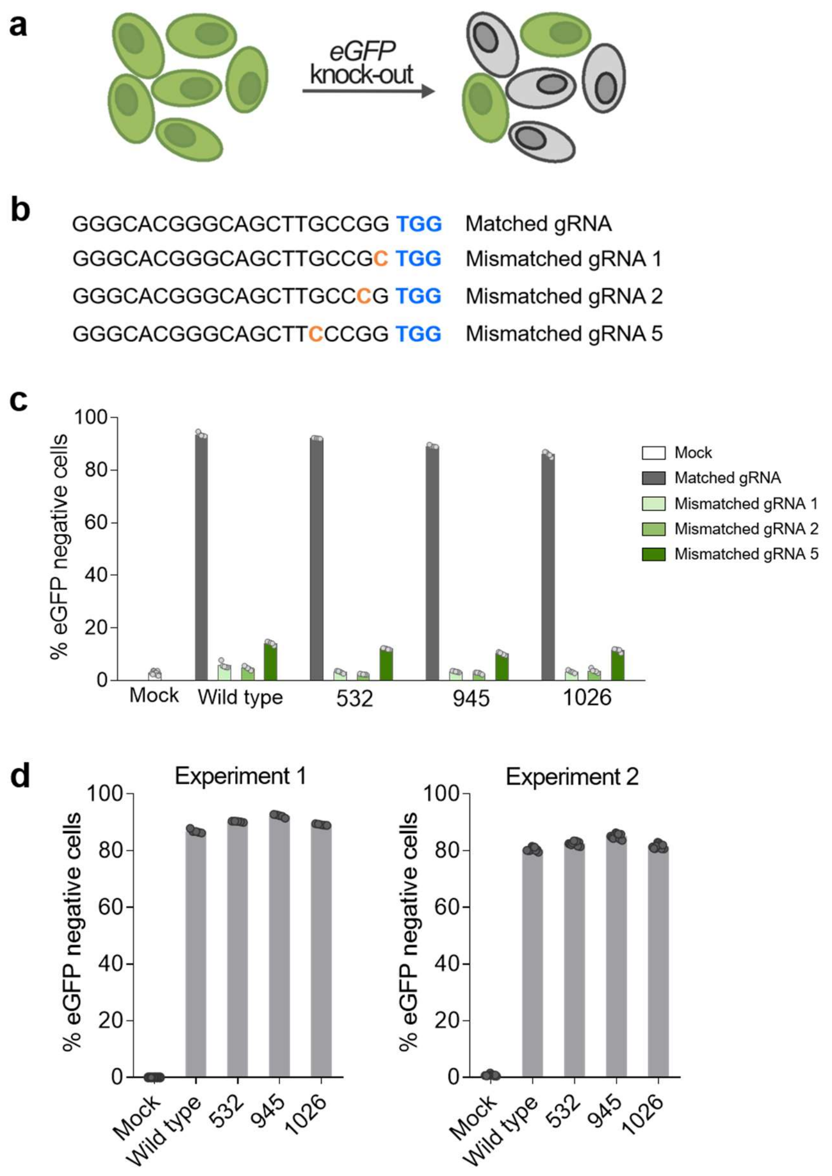

Supplementary Figure 4. *eGFP* knock-out assay. (a) Schematic of the *eGFP* knock-out assay to investigate the off-target profile of the Cas9-adaptor conjugates. The *eGFP·PEST* gene stably expressed in U2OS cells is targeted by Cas9 RNP using matched and mismatched gRNAs. (b) Sequences of the gRNAs. Mismatch sites are in orange. PAM sequences are highlighted in blue. (c) Results of the *eGFP* knock-out assay. Cells were nucleofected with 10 pmol of RNP and were incubated for 48 h followed by nuclei staining and fluorescence imaging. Unlabeled wild type Cas9 and Cas9-adaptors labeled at the indicated residues were used (n = 14 technical replicates for mock, and n = 5 technical replicates for other conditions). (d) Results of the *eGFP* knock-out assay using Cas9-PEG conjugates. The same procedures as in (c) were employed. Results from two independent experiments are shown, with either 5 technical replicates (experiment 1) or 10 technical replicates (experiment 2). Source data are provided as a Source Data file.

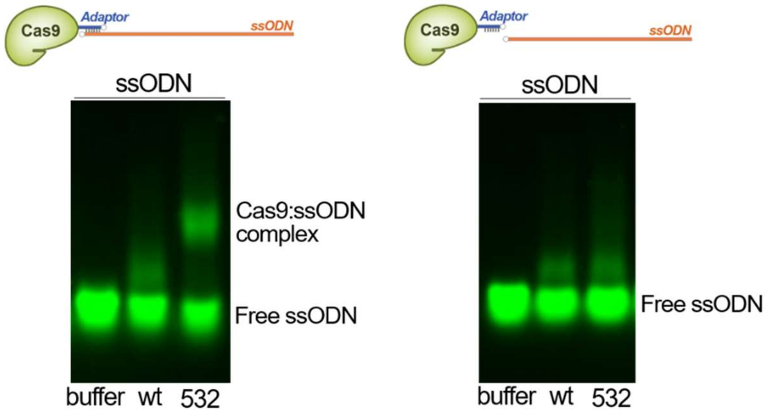

Supplementary Figure 5. Electrophoretic mobility shift assay to check the binding between Cas9-adaptor conjugates and ssODN. When the ssODN contained the adaptor-binding sequence, the specific Cas9:ssODN complex was observed. In contrast, only non-specific binding patterns were observed when the ssODN did not have the corresponding sequence or when the unlabeled Wild type Cas9 (wt) was used. The ssODN for *HiBiT* knock-in at the *GAPDH* locus was used. Even though the lanes are not contiguous between wt and 532, they are all from a single gel. Representative results from two independent experiments are shown.

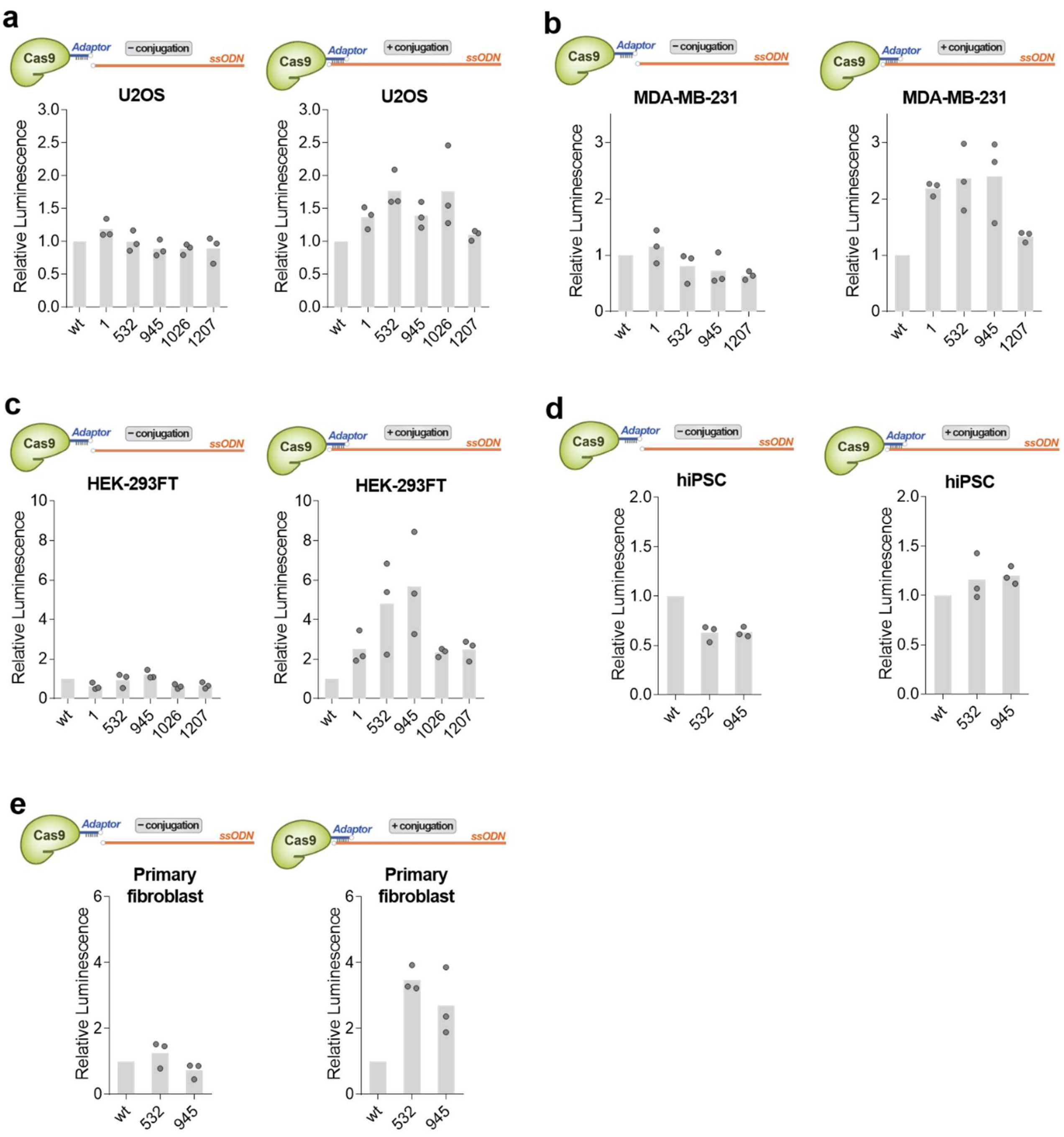

Supplementary Figure 6. *HiBiT* knock-in results in multiple cell types. (a-e) *HiBiT* knock-in results in (a) U2OS cells, (b) MDA-MB-231 cells, (c) HEK-293FT cells, (d) human induced pluripotent stem cells, and (e) primary human neonatal dermal fibroblasts. The panels on the left show luminescence intensities using the separate Cas9/ssODN system. The panels on the right show luminescence intensities from Cas9:ssODN conjugates. Unlabeled wild type Cas9 (wt) and Cas9-adaptors labeled at the indicated residues were used (n = 3 biologically independent experiments). Source data are provided as a Source Data file.

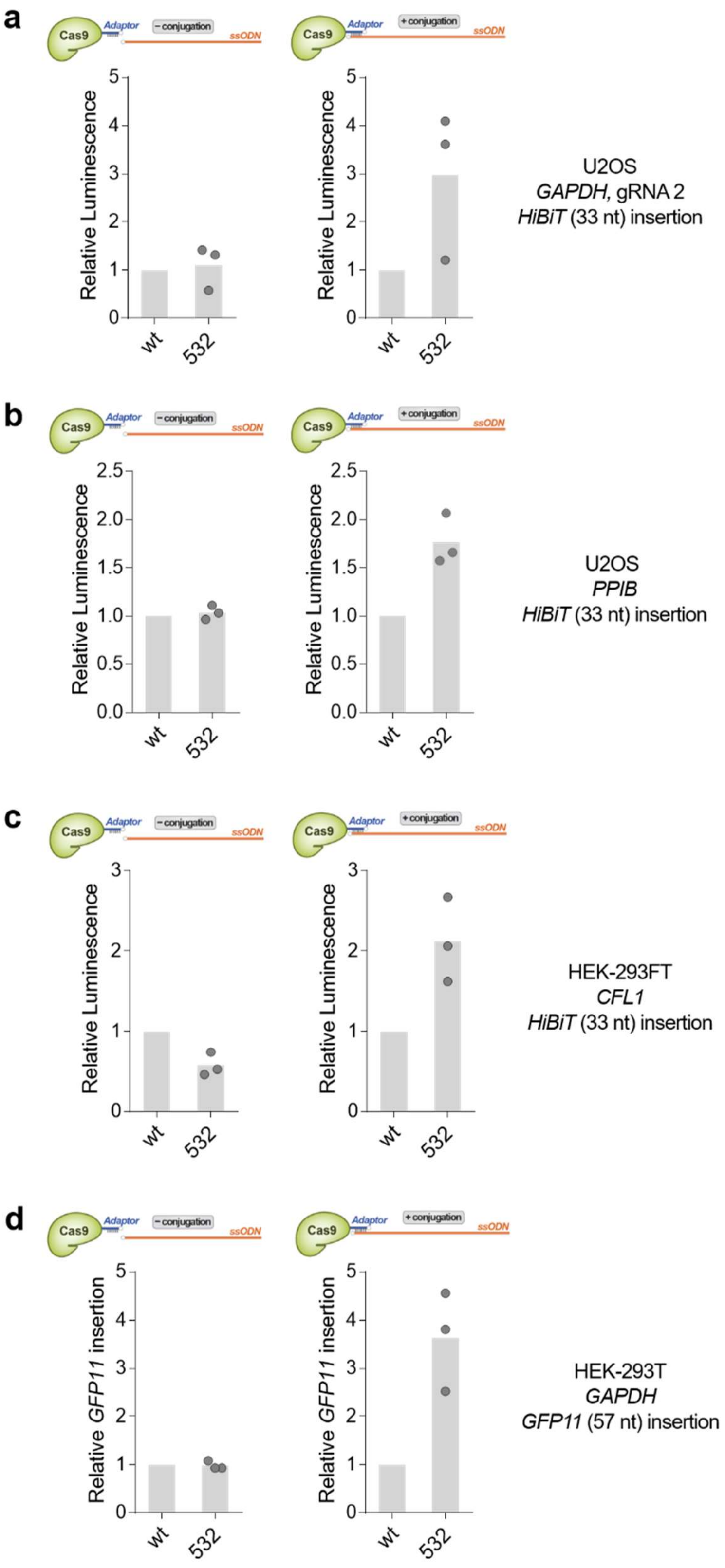

Supplementary Figure 7. Genome editing results in multiple conditions. (a) Another *GADPH*-targeting gRNA was used for *HiBiT* knock-in. (b) *PPIB* locus was targeted for *HiBiT* knock-in. (c) *CFL1* locus was targeted for *HiBiT* knock-in. (d) The *GFP11* sequence was inserted at the *GAPDH* locus. Either a separate Cas9/ssODN system (left panels) or a Cas9:ssODN conjugate (right panels) was used. Unlabeled wild type Cas9 (wt) and Cas9-adaptor labeled at residue 532 were used (n = 3 biologically independent experiments). Source data are provided as a Source Data file.

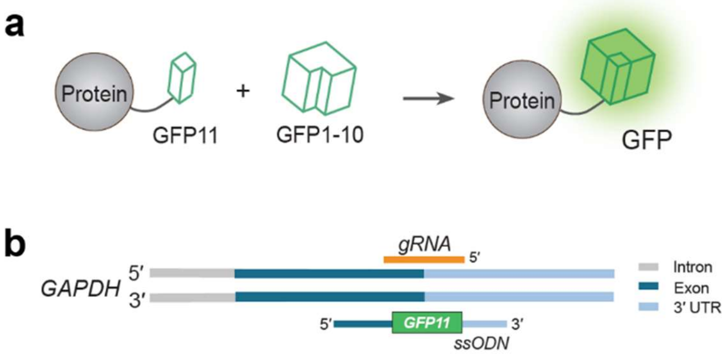

Supplementary Figure 8. GFP complementation assay to check the HDR-mediated insertion of the 57-nt *GFP11* fragment. (a) *GFP11* sequence knock-in right before the stop codon of the gene of interest results in the expression of a fusion protein having a C-terminal GFP11 tag. When the other fragment of GFP (GFP1-10) is supplied, a fully functional GFP is reconstituted, and the fluorescence signal can be detected.[3,4] (b) Design strategy for *GFP11* knock-in at the *GAPDH* locus.

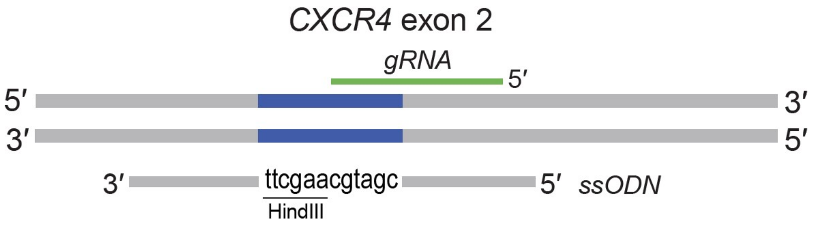

Supplementary Figure 9. HDR-mediated 12-base exchange on exon 2 of *CXCR4* introduces a HindIII restriction site from which the HDR efficiency can be measured.

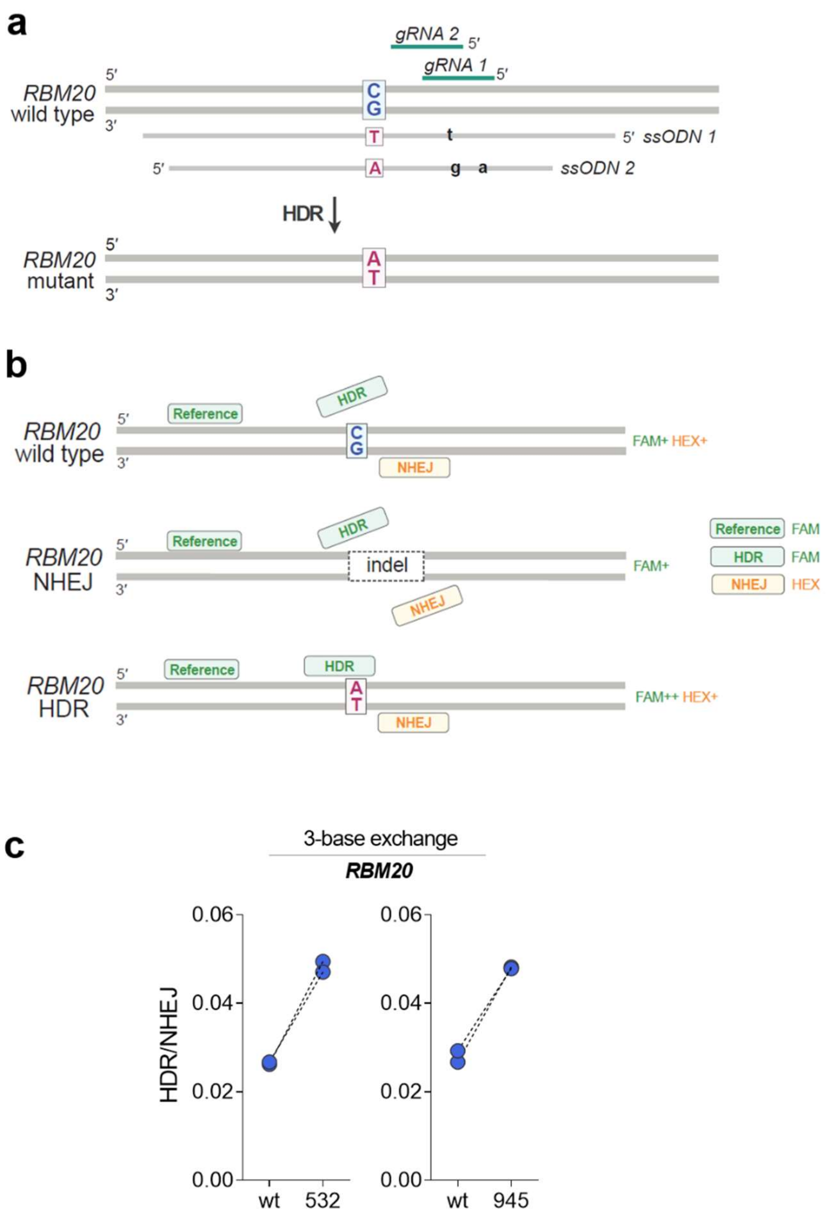

Supplementary Figure 10. Droplet digital PCR-based quantification of nucleotide exchange at the *RBM20* locus. (a) One of the CG pair is replaced by AT pair. Additional silent mutations introduced by ssODNs are shown in black lowercase letters. (b) Schematic of the droplet digital PCR-based quantification of NHEJ and HDR.[5,6] The reference probe can bind to all alleles while the HDR probe binds only to the precisely edited allele. The NHEJ probe is a drop-off probe that cannot bind to the NHEJ-repaired allele. Each probe is labeled with a fluorophore-quencher pair. During the PCR, DNA-bound probes are hydrolyzed by the exonuclease activity of the DNA polymerase. Therefore, fluorophores and quenchers move apart from each other, providing fluorescence signals. (c) Three-base exchange at the *RBM20* locus was promoted by Cas9:ssODN conjugates in HEK-293FT cells. Unlabeled wild type Cas9 (wt) and Cas9-adaptor labeled at the indicated residues were used. gRNA 2 and ssODN 2 were used (n = 2 biologically independent experiments). Source data are provided as a Source Data file.

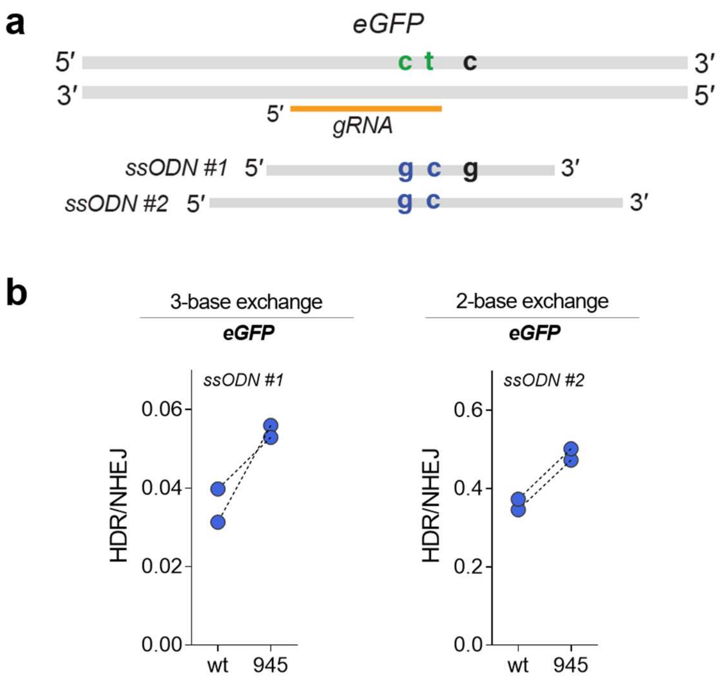

Supplementary Figure 11. Conversion of *eGFP* to *BFP*. (a) HDR-mediated 2-base exchange (c to g and t to c, shown in green and blue) coverts e*GFP* to *BFP*. ssODN #1 induces the 2-base exchange and introduces an extra silent mutation (c to g, shown in black). ssODN #2 induces the 2-base exchange, and has longer homology arms. (b) The e*GFP* to *BFP* conversion efficiency was increased by Cas9-ssODN conjugation in U2OS cells stably expressing *eGFP.PEST* (n = 2 biologically independent experiments). Source data are provided as a Source Data file.

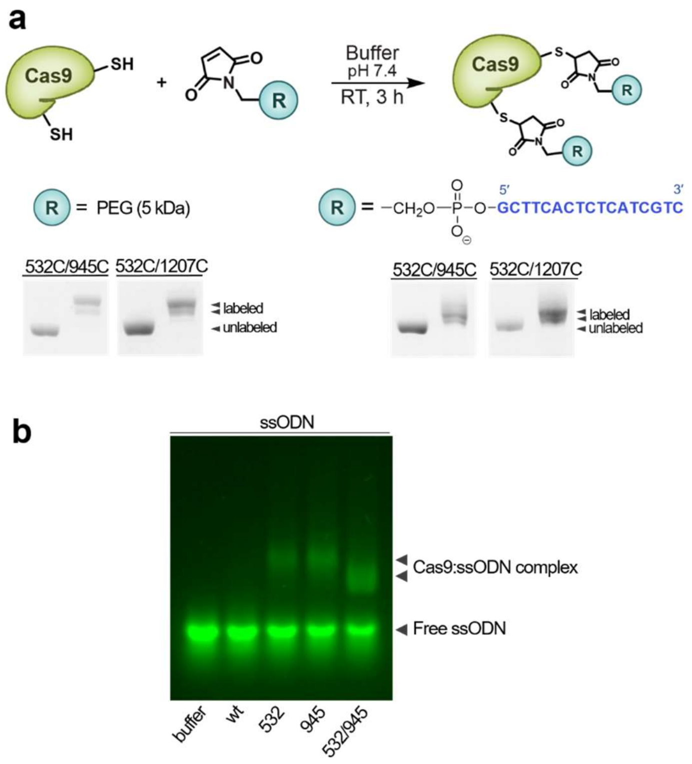

Supplementary Figure 12. Multivalent display of ssODN. (a) Site-specific labeling of Cas9 mutants at two cysteine residues using thiol-maleimide conjugation. The degree of labeling was measured through SDS-PAGE followed by Coomassie staining. The images show results from single experiments (left, PEG labeling) or representative results from two independent experiments (right, DNA labeling). Full-gel images are provided as a Source Data file. (b) An electrophoretic mobility shift assay was performed using Cas9-adaptors and the ssODN specific for *GAPDH HiBiT* tagging that contained the adaptor-binding sequence. Unlabeled wild type Cas9 (wt) and Cas9-adaptors labeled at the indicated residues were used. The RNP and ssODN were used at a molar ratio of 1:2. Representative results from two independent experiments are shown.

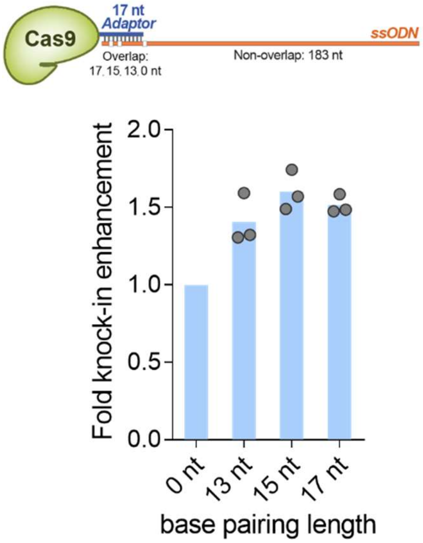

Supplementary Figure 13. Effect of the base-pairing length on the HDR-enhancing capability of the Cas9:ssODN conjugate. *HiBiT* sequence insertion was employed as a test HDR assay in U2OS cells using the Cas9-adaptor labeled at residue 945 (n = 3 biologically independent experiments). Shorter hybridization length was tested to ensure that the adaptor attachment does not interfere with Cas9 function and make chemical labeling easier. Up to 13-nt hybridization was tested, because the 13-nt base pairing had a predicted melting temperature substantially higher than 37°C under the physiological conditions. Thus, it is assumed that 13-nt pairing would have a sufficient specificity for binding between the adaptor and ssODN. Source data are provided as a Source Data file.

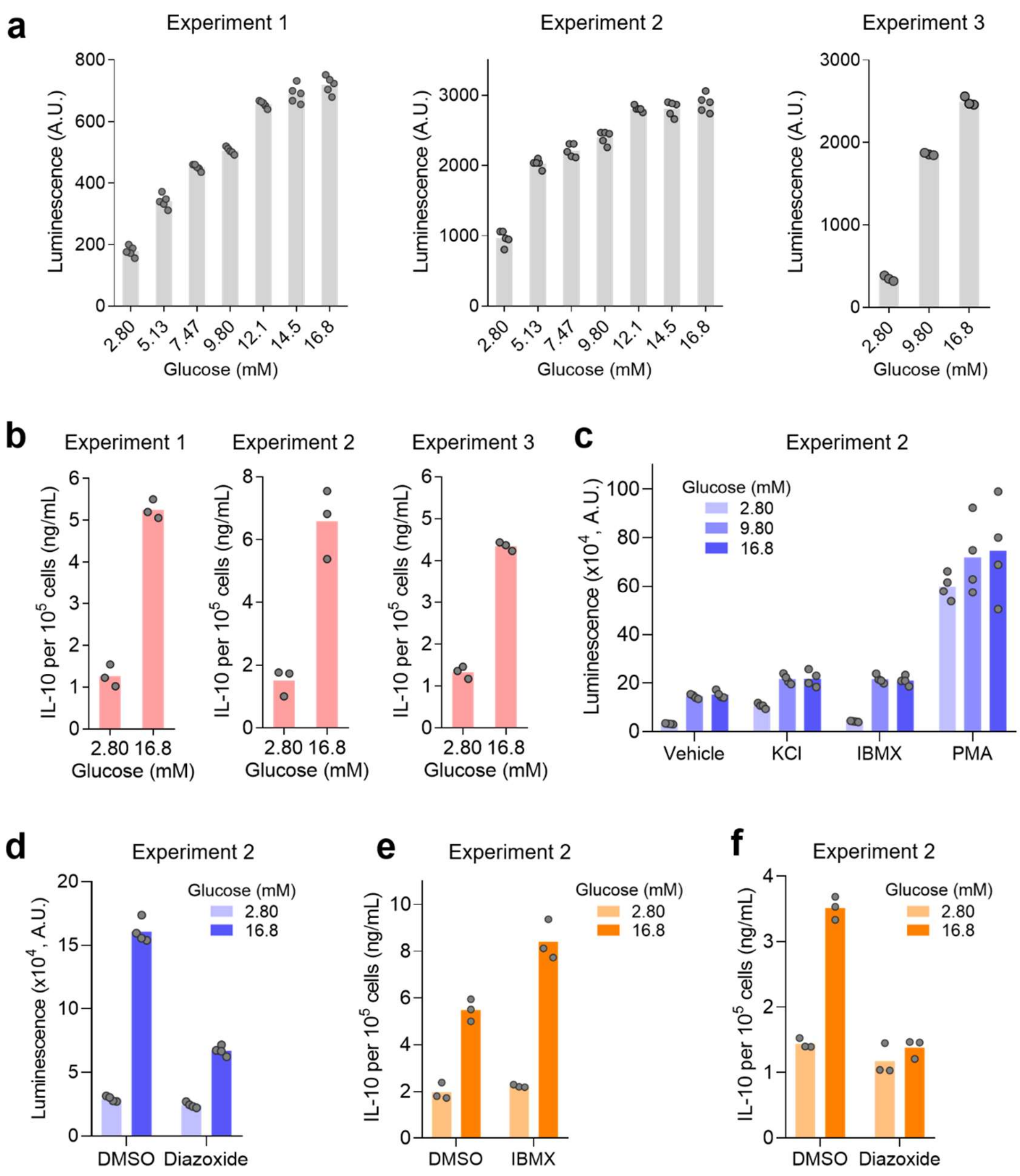

Supplementary Figure 14. Secretion of HiBiT peptide and IL-10 from edited INS-1E cells. (a) Glucose-stimulated HiBiT peptide secretion from edited INS-1E cells. Results from three independent experiments are shown, with 5 technical replicates for experiment 1 and experiment 2, and 3 technical replicates for experiment 3. (b) Glucose-stimulated IL-10 secretion from edited INS-1E cells. Results from three independent experiments are shown, each with 3 technical replicates. (c-d) Effect of (c) known insulin secretagogues and (d) diazoxide on the HiBiT peptide secretion (n = 4 technical replicates). (e-f) Effect of (e) IBMX and (f) diazoxide on the IL-10 secretion (n = 3 technical replicates). IBMX, 3-isobutyl-1-methylxanthine. PMA, phorbol 12-myristate 13-acetate. A.U., Arbitrary unit. Source data are provided as a Source Data file.

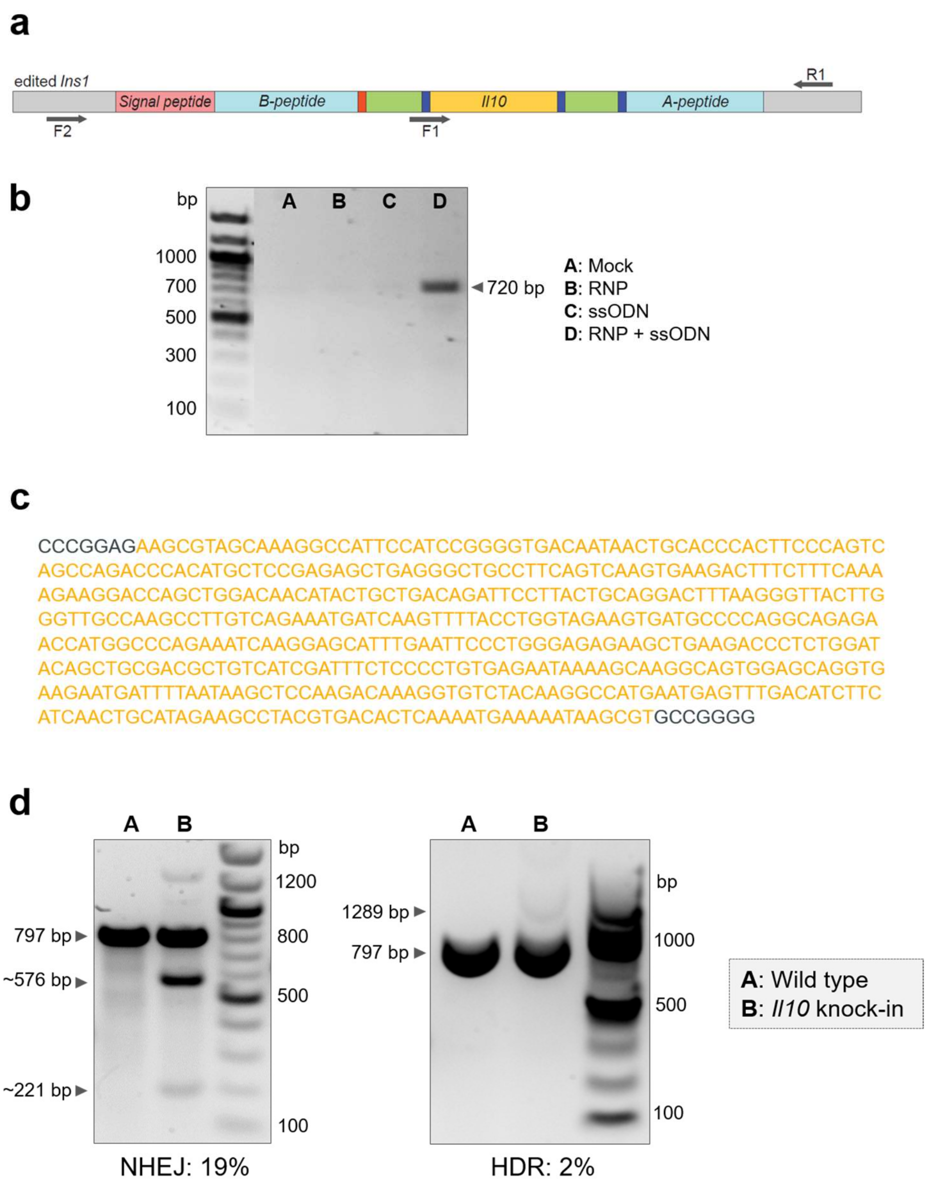

Supplementary Figure 15. Confirmation of *Il10* knock-in by PCR. (a) Schematic of the PCR where the knock-in-specific (F1) and universal (F2, R1) primers were used. (b) Genomic DNA was extracted from cells 72 h after transfection, and PCR was performed followed by agarose gel electrophoresis and ethidium bromide staining. Primers F1 and R1 were used. Even though the lanes are not contiguous between the maker and sample A, they are all from a single gel. The result of a single genotyping experiment is shown. (c) *Il10* knock-in sequence confirmed by Sanger sequencing. Yellow sequence indicates the knock-in region. (d) Estimation of the degree of NHEJ and the *Il10* insertion from the knock-in-enriched cells. PCR was performed using the primers F2 and R1, followed by T7E1 assay or direct agarose gel electrophoresis and SYBR gold staining. Quantification of the band intensity shows 19% of NHEJ and 2% of the *Il10* knock-in at *ins1* gene. Results of a single experiment is shown.

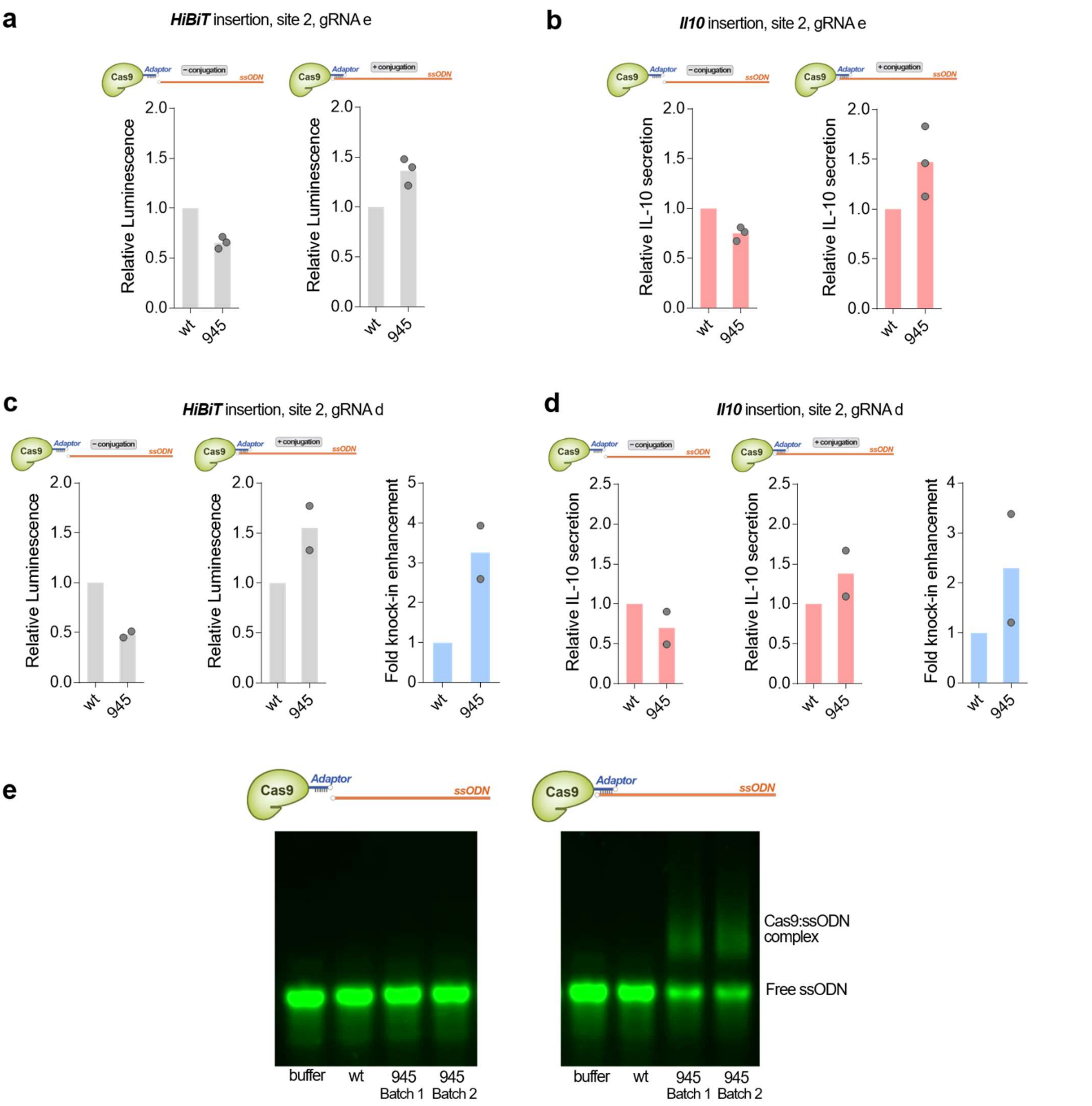

Supplementary Figure 16. Cas9:ssODN conjugate enhanced precision genome editing in INS-1E cells. (a–d) Both *HiBiT* knock-in and *Il0* knock-in were promoted by Cas9-ssODN conjugation when two different gRNAs were tested. Unlabeled wild type Cas9 (wt) and Cas9-adaptor labeled at residue 945 were used (n = 3 biologically independent experiments for panels a and b, and n = 2 biologically independent experiments for panels c and d). Source data are provided as a Source Data file. (e) Electrophoretic mobility shift assay to check the binding between Cas9-adaptor and long ssODNs for *Il0* knock-in. The specific Cas9:ssODN complex was observed only when both Cas9 and ssODN contained the complementary adaptor sequences. All lanes are from a single gel. Unlabeled wild type Cas9 (wt) and Cas9-adaptor labeled at residue 945 from different batches were used. Representative results from two independent experiments are shown.

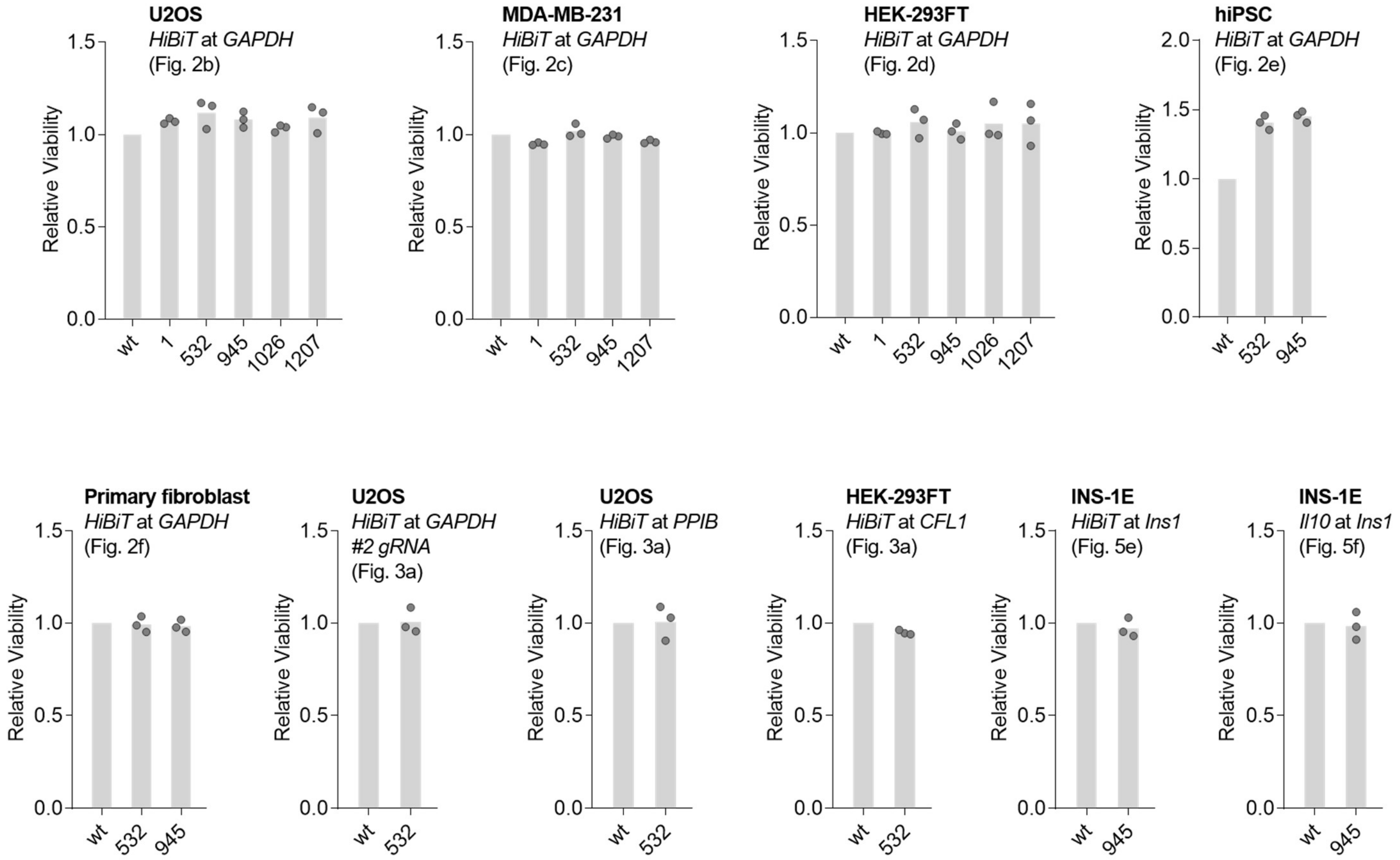

Supplementary Figure 17. Cell viabilities after genome editing. The viabilities were quantified using the PrestoBlue reagent by measuring the conversion of resazurin to fluorescent resorufin. Cell types and corresponding figure numbers are shown on top of the plots. Unlabeled wild type Cas9 (wt) and Cas9-adaptors labeled at the indicated residues were used with the Cas9:ssODN conjugation system (n = 3 biologically independent experiments). Source data are provided as a Source Data file.

2. Supplementary References

1 Jinek, M. *et al.* Structures of Cas9 endonucleases reveal RNA-mediated conformational activation. *Science* **343**, 1247997 (2014).

2 Jiang, F. G., Zhou, K. H., Ma, L. L., Gressel, S. & Doudna, J. A. A Cas9-guide RNA complex preorganized for target DNA recognition. *Science* **348**, 1477–1481 (2015).

3 Kamiyama, D. *et al.* Versatile protein tagging in cells with split fluorescent protein. *Nat. Commun.* **7**, 11046 (2016).

4 Leonetti, M. D., Sekine, S., Kamiyama, D., Weissman, J. S. & Huang, B. A scalable strategy for high-throughput GFP tagging of endogenous human proteins. *P. Natl. Acad. Sci. USA* **113**, E3501–E3508, (2016).

5 Miyaoka, Y. *et al.* Systematic quantification of HDR and NHEJ reveals effects of locus, nuclease, and cell type on genome-editing. *Sci. Rep.* **6**, 23549 (2016).

6 Kato-Inui, T., Takahashi, G., Hsu, S. & Miyaoka, Y. Clustered regularly interspaced short palindromic repeats (CRISPR)/CRISPR-associated protein 9 with improved proof-reading enhances homology-directed repair. *Nucleic Acids Res.* **46**, 4677–4688 (2018).
