# Supplementary material for: Engineering designer beta cells with a CRISPR-Cas9 conjugation platform
Source: Nat Commun. 2020 Aug 13;11:4043. doi: 10.1038/s41467-020-17725-0 (PMC7426819; doi:10.1038/s41467-020-17725-0)
Supplement: Supplementary file 2 — Reporting Summary [file 41467_2020_17725_MOESM2_ESM.pdf]

nature research

Corresponding author(s): Amit Choudhary

Last updated by author(s): Jun 25, 2020

Reporting Summary

Nature Research wishes to improve the reproducibility of the work that we publish. This form provides structure for consistency and transparency in reporting. For further information on Nature Research policies, see Authors & Referees and the Editorial Policy Checklist .

Statistics

For all statistical analyses, confirm that the following items are present in the figure legend, table legend, main text, or Methods section.

n/a	Confirmed	
☐	☒	The exact sample size (*n*) for each experimental group/condition, given as a discrete number and unit of measurement
☐	☒	A statement on whether measurements were taken from distinct samples or whether the same sample was measured repeatedly
☐	☒	The statistical test(s) used AND whether they are one- or two-sided<br>*Only common tests should be described solely by name; describe more complex techniques in the Methods section.*
☒	☐	A description of all covariates tested
☐	☒	A description of any assumptions or corrections, such as tests of normality and adjustment for multiple comparisons
☐	☒	A full description of the statistical parameters including central tendency (e.g. means) or other basic estimates (e.g. regression coefficient) AND variation (e.g. standard deviation) or associated estimates of uncertainty (e.g. confidence intervals)
☐	☒	For null hypothesis testing, the test statistic (e.g. *F*, *t*, *r*) with confidence intervals, effect sizes, degrees of freedom and *P* value noted<br>*Give P values as exact values whenever suitable.*
☒	☐	For Bayesian analysis, information on the choice of priors and Markov chain Monte Carlo settings
☒	☐	For hierarchical and complex designs, identification of the appropriate level for tests and full reporting of outcomes
☒	☐	Estimates of effect sizes (e.g. Cohen's *d*, Pearson's *r*), indicating how they were calculated

Our web collection on statistics for biologists contains articles on many of the points above.

Software and code

Policy information about availability of computer code

Data collection	PerkinElmer EnVision Manager 1.13, Molecular Devices MetaXpress, PerkinElmer Operetta Harmony 4.8, Molecular Devices SoftMax Pro 7.0, Azure Biosystems Azure C600, BioRad QuantaSoft 1.6.6
Data analysis	GraphPad Prism 8, Microsoft Excel 2016, Molecular Devices MetaXpress, PerkinElmer Operetta Harmony 4.8, ImageJ 1.52a, BioRad QuantaSoft 1.6.6, UCSF Chimera 1.13

For manuscripts utilizing custom algorithms or software that are central to the research but not yet described in published literature, software must be made available to editors/reviewers. We strongly encourage code deposition in a community repository (e.g. GitHub). See the Nature Research guidelines for submitting code & software for further information.

Data

Policy information about availability of data

All manuscripts must include a data availability statement. This statement should provide the following information, where applicable:

- Accession codes, unique identifiers, or web links for publicly available datasets
- A list of figures that have associated raw data
- A description of any restrictions on data availability

The data generated in this study are provided in the manuscript and the supplementary information, and are available from the corresponding author upon reasonable request. The source data for Figures 2–6 and Supplementary Figures 2, 4, 6, 7, 10–14, 16, and 17 are provided as a Source Data file. Plasmids from Addgene (#98158 [https://www.addgene.org/98158], and #70219 [https://www.addgene.org/70219]) were used in this study. Structural information from PDB (ID: 5F9R [https://www.rcsb.org/structure/5F9R], 4CMP [https://www.rcsb.org/structure/4CMP], and 4ZT0 [https://www.rcsb.org/structure/4ZT0]) was used in this study.

Field-specific reporting

Please select the one below that is the best fit for your research. If you are not sure, read the appropriate sections before making your selection.

☒ Life sciences ☐ Behavioural & social sciences ☐ Ecological, evolutionary & environmental sciences

For a reference copy of the document with all sections, see nature.com/documents/nr-reporting-summary-flat.pdf

Life sciences study design

All studies must disclose on these points even when the disclosure is negative.

Sample size	Sample sizes (number of biological replicates for cellular experiments) were chosen based on previous literatures about genome editing (e.g., Nature 517, 583–588 / Nat. Biotechnol. 34, 339–344).
Data exclusions	No data were excluded from the analyses.
Replication	At least two biological replicates were used for cellular experiments except for the experiment in figure 6e. Protein–DNA conjugates used for cellular experiments were independently prepared at least twice. Exact replicate numbers for each experiment are provided at figure legends. All attempts at replication were successful.
Randomization	Cell cultures, treatments, and measurements were performed under identical conditions; No randomization was applied.
Blinding	Cell cultures, treatments, and measurements were performed under identical conditions; No blinding was applied.

Reporting for specific materials, systems and methods

We require information from authors about some types of materials, experimental systems and methods used in many studies. Here, indicate whether each material, system or method listed is relevant to your study. If you are not sure if a list item applies to your research, read the appropriate section before selecting a response.

Materials & experimental systems

n/a	Involved in the study	
☒	☐	Antibodies
☐	☒	Eukaryotic cell lines
☒	☐	Palaeontology
☒	☐	Animals and other organisms
☒	☐	Human research participants
☒	☐	Clinical data

Methods

n/a	Involved in the study	
☒	☐	ChIP-seq
☒	☐	Flow cytometry
☒	☐	MRI-based neuroimaging

Eukaryotic cell lines

Policy information about cell lines

Cell line source(s)	U2OS.eGFP.PEST cells were obtained from Keith Joung's Lab. HEK-293T cells were obtained from ATCC. HEK-293FT cells were obtained from Feng Zhang's lab. MDA-MB-231 cells were obtained from Stuart Schreiber's lab. Human induced pluripotent stem cells were obtained from Gibco (A18945). Primary human dermal neonatal fibroblast cells were obtained from Gibco (C0045C). INS-1E cells were obtained from Bridget Wagner's lab.
Authentication	None of the cell lines were authenticated.
Mycoplasma contamination	All the cell lines except primary fibroblast were routinely checked for Mycoplasma contamination using Universal Mycoplasma Detection Kit (ATCC, # 30-1012K). All the cell lines were Mycoplasma-negative. The primary fibroblast was confirmed to be Mycoplasma-negative by the supplier, and used directly after receiving.
Commonly misidentified lines (See ICLAC register)	No commonly misidentified cell lines were used.
